# A numerical frame work of magnetically driven Powell-Eyring nanofluid using single phase model

**DOI:** 10.1038/s41598-021-96040-0

**Published:** 2021-08-13

**Authors:** Wasim Jamshed, Mohamed R. Eid, Kottakkaran Sooppy Nisar, Nor Ain Azeany Mohd Nasir, Abhilash Edacherian, C. Ahamed Saleel, V. Vijayakumar

**Affiliations:** 1 Department of Mathematics, Capital University of Science and Technology (CUST), Islamabad, 44000 Pakistan; 2 Department of Mathematics, Faculty of Science, New Valley University, Al-Kharga, Al-Wadi Al-Gadid 72511 Egypt; 3 Department of Mathematics, Faculty of Science, Northern Border University, Arar, 1321 Saudi Arabia; 4 Department of Mathematics, College of Arts and Sciences, Prince Sattam Bin Abdulaziz University, Wadi Aldawaser, 11991 Saudi Arabia; 5 Department of Mathematics, Centre for Defence Foundation Studies, Universiti Pertahanan Nasional Malaysia, Kem Sungai Besi, 57000 Kuala Lumpur, Malaysia; 6 Department of Mechanical Engineering, College of Engineering, King Khalid University, Asir-Abha, 61421 Saudi Arabia; 7 Department of Mathematics, School of Advanced Sciences, Vellore Institute of Technology, Vellore, India

**Keywords:** Mathematics and computing, Physics

## Abstract

The current investigation aims to examine heat transfer as well as entropy generation analysis of Powell-Eyring nanofluid moving over a linearly expandable non-uniform medium. The nanofluid is investigated in terms of heat transport properties subjected to a convectively heated slippery surface. The effect of a magnetic field, porous medium, radiative flux, nanoparticle shapes, viscous dissipative flow, heat source, and Joule heating are also included in this analysis. The modeled equations regarding flow phenomenon are presented in the form of partial-differential equations (PDEs). Keller-box technique is utilized to detect the numerical solutions of modeled equations transformed into ordinary-differential equations (ODEs) via suitable similarity conversions. Two different nanofluids, Copper-methanol (Cu-MeOH) as well as Graphene oxide-methanol (GO-MeOH) have been taken for our study. Substantial results in terms of sundry variables against heat, frictional force, Nusselt number, and entropy production are elaborate graphically. This work’s noteworthy conclusion is that the thermal conductivity in Powell-Eyring phenomena steadily increases in contrast to classical liquid. The system’s entropy escalates in the case of volume fraction of nanoparticles, material parameters, and thermal radiation. The shape factor is more significant and it has a very clear effect on entropy rate in the case of GO-MeOH nanofluid than Cu-MeOH nanofluid.

## Introduction

The first to introduce the theory of the boundary layer was Ludwig Prandtl^1^. A boundary-layer is the tinny region of fluid flow in which flow is influenced by the friction between the solid plate and the liquid. The boundary layer flow has been broadly deliberated in the literature and plays a vital role in fluid dynamics. The investigation of boundary-layer flowing past a horizontal plate had countless manufacturing implementations, such as food manufacturing, glass fibers production, manufacturing of rubber sheets, extrusion, metal spinning, wire drawing, and cooling of massive metallic plates such as an electrolyte^2–4^. Makinde and Onyejekwe^5^ presented the numerical computations for the boundary-layer flowing model results in the stretching sheet with variable electrical conductivity and variable viscosity using a shooting technique and a sixth-order RK integration algorithm. They concluded that, when the electrical conductivity parameter is increased, convective heat transfer and skin friction coefficient decreases within the boundary surface.

Moreover, a rise in the variable viscosity parameter increases viscous force and makes viscous forces dominant over the applied magnetic field. In the use of numerical shootings, Ibrahim and Makinde^6^ looked at the boundary-layer movement past a vertical, rotating flat sheet with heating effects and chemical reaction effects from Joule. Heat transmission is the thermal energy transfer from one device to another because of temperature differences. Because of this temperature difference, the heat transmission process takes place between two bodies (or a related body). In many industrial applications such as composite materials manufacturing, geothermal reservoirs, porous solid drying, thermal isolation, oil recovery, and the transport of sub-terrain species, the research into fluid flows and heat transmission produced by stretching media is of great importance. In the above situations, heat transfer and flow assessment are important as the final product efficiency is calculated based on the velocity gradient (skin friction) coefficient and the convective heat shift rate. Elbashbeshy^7^ numerically studied viscous fluid and heat transfer flow by assuming the exponentially continuous stretching sheet. In his work, fluid inhabits the distance over an endless horizontal plate, and the nonlinear extending of the plate induces the flow. He implements the numerical technique to solve the modeled equations. The results indicated that the suction parameter could cool the continuous moving stretching surface. The numerical results also showed that the thermal boundary layer’s thickening level reduces for larger values of the suction parameter. After that, Sanjayanand and Khan^8^ prolonged the work of Elbashbeshy^7^ to provide the heat and mass transfer, nonlinear expanding, of second-order viscoelastic fluid. The results of their work are elastic deformation and viscidness dissipative flow. The key conclusions reached by the authors were that with increased local viscoelastic parameters the speed gradient and convective heat exchange (Nusselt number) fall on the frontier surface. Magyari and Keller^9^ developed numerical findings for mass transmission and viscous fluids due to an expanding layer. The readers can research the fluid flow and heat transfer qualities of the borderline layer on a moving surface^10–12^.

Keeping given the importance of heat transport phenomena results in fluid flowing in thermal devices, Choi^13^ introduced the nanofluids’ concept by including solid additive (nanoparticles) having a size of less than $100$ nm in the conventional liquids. The nanoparticles are usually made of metals and their oxides, nitrides, carbides, etc. The metallic particles enhance heat conduction properties of the base liquids such as water (H_2_O), methanol (MeOH) and ethylene glycol (EG), etc. Since the nanofluids tend to increase the heat transfer rate, they have applications in industrial processes like the coolant in nuclear reactors, heat flowing controllers in heat valves, radiators of cars, and frontal vehicle temperature. The cooling and heating of water with nanofluids can preserve trillion Btu of energy^14^. The power of nanofluids to heat allows the computer processors to cool down. Cancer can be treated with medications and radiation in medical sciences with iron-based nanofluids^15–17^. Eastman et al.^18^ pondered the thermal conductivity phenomenon regarding Cu-Ethylene glycol (EG) nanofluids and found that the ethylene glycol’s thermal conductivity improves 40% after the addition of 0.3 vol % of average diameter, 10 nm nanoparticles in the base fluid. Nanofluids, particularly heat transfer and boundary layer flow, are being extensively investigated and studied. Wang et al.^19^ and Keblinski et al.^20^ presented a thorough review of the literature. In the wetting, propagation, and dispersion capacity on the surface of solid compared to conventional fluids, Buongiorno^21^ found nanofluids had greater stability. Recent additions to heat and mass transfer nanofluids can find in^22–26^.

Nanofluids may in some circumstances be non-Newtonian, even viscoelastic. Additional experimental researches are necessary to establish nanofluid viscosity models for use in simulation studies^27, 28^. For these reasons, we regard the fluid Powell-Eyring, which is presented here and taking into consideration the significance of non-Newtonian fluids. Two investigators Powell and Eyring^29^ proposed this hypothesis in 1944. Also, a type of visco-elastic fluid is the Powell-Eyring. Eyring–Powell somehow introduces a more complicated mathematical framework, but offers some advantages over previous viscoelastic fluid models. This model does not come from empirical expressions as most models base the kinetic theories of liquids. This model likewise has the Newtonian properties at low and great shear stress. Examples of Powell-Eyring fluids contain polymer melts and suspensions of solids in non-Newtonian liquids. This non-Newtonian Powell-Eyring fluid has many implementations like these are utilized in many engineering, manufacturing, and industrial areas such as polymers, pulp, plasma, and other biological technology. However, several researchers have investigated the non-Newtonian Powell-Eyring nanofluid behavior, such as^30–32^. Aziz and Afy^33^ used the shooting technique to get the Casson nanofluid’s numerical solution over a stretching sheet using the Buongiorno nanofluid model. They concluded that Hall parameter upsurge in the convective rate of heat and mass transfer and the drag coefficient for initial stages of flow, i.e., primary and secondary flow. Moreover, for growing velocity slip values, the nanoparticle volume concentration parameter increases and reduces the Sherwood number. Ali et al.^34^ investigated that, with the modification of Fourier's law, the influences on the magnet field of Dufour and Soret travel via an extended sheet with non-rotational Newton's Oldroyd-B nanofluid stream. To calculate the numerical findings, the Galerkin-Finite element system was utilized. They concluded that the concentration of the nanoparticles decreased against the parameter of thermal-relaxation, Soret, and Lewis. At the same time, the magnetic field and Deborah's number raise the temperature profile. In addition, at increasing Brownian and rotational values the heat transmission rate is decreasing. Then, Abdelmalek et al.^35^ did a thorough examination of the nanofluid moving via varied stretchings by the shooting technique of Joule heating and thermal radiation. The researchers noticed that the magnitude of the mass transfer rate is minimal with large Lewis values, while the Prandtl number has an important influence. Furthermore, Kebede et al.^36^ launched an investigation of the electrically conductive flow carried out by nanofluid Williamson in the heat source and the chemically reactive species. In the existence of Brownian diffusion, Gireesha et al.^37^ recently evaluated Jeffrey nanofluid's 3-D boundary-layer flow via a porous stretched plate. Their work was based on a two-phase nanofluid model and the number of findings was calculated via the RK-4 approach. Notable findings have recently been reported on non-Newtonian nanofluids, see for example^38–41^.

In general, entropy represents system disorder. The system disorder’s meaning is known as the system’s inability to utilize the useful energy of 100%. When the energy of the system conserves perfectly, entropy becomes zero, but this is not the case in the actual world. The entropy of the system is increasing all the time. Many researchers across the world have studied to invent innovative ideas regarding entropy minimization due to its vast utilization in the industry. Sheikholeslami et al.^42^ studied magneto nanofluid flow past an expandable surface with entropy generation phenomenon. While Abolbashari et al.^43^ contemplated entropy generation in Casson nanofluids flow. The axisymmetric fluid flow towards a time-dependent radially expandable plate was explored by Shahzad et al.^44^. Similar investigation is conducted on nanofluid entropy production with expanded surfaces having various shapes^45, 46^.

According to the prior researches, fluid flow and heat transport for Cu-MeOH and GO-MeOH as non-Newtonian nanofluidic are not investigated. The non-Newtonian nanofluid has to be investigated since it mitigates global warming and provides a clean alternative energy source. This paper therefore develops, past an expanded uniform surface, a streamlined mathematical model for the boundary-layer fluid flow. The Powell-Eyring nanofluid model is considered as operating fluid embedded with slip as well as convective boundary conditions. Furthermore, thermal radiation, viscous dissipation, heat source, and Joule heating are included for heat transfer analysis with the consideration of Cu-MeOH and GO-MeOH as base nanoliquids. The obtained consequences are plotted against velocity, temperature ($T$), entropy distributions, the surface drag ${C}_{f}$, and heat transfer phenomenon $Nu$.

## Mathematical formulation

**Figure 1:**
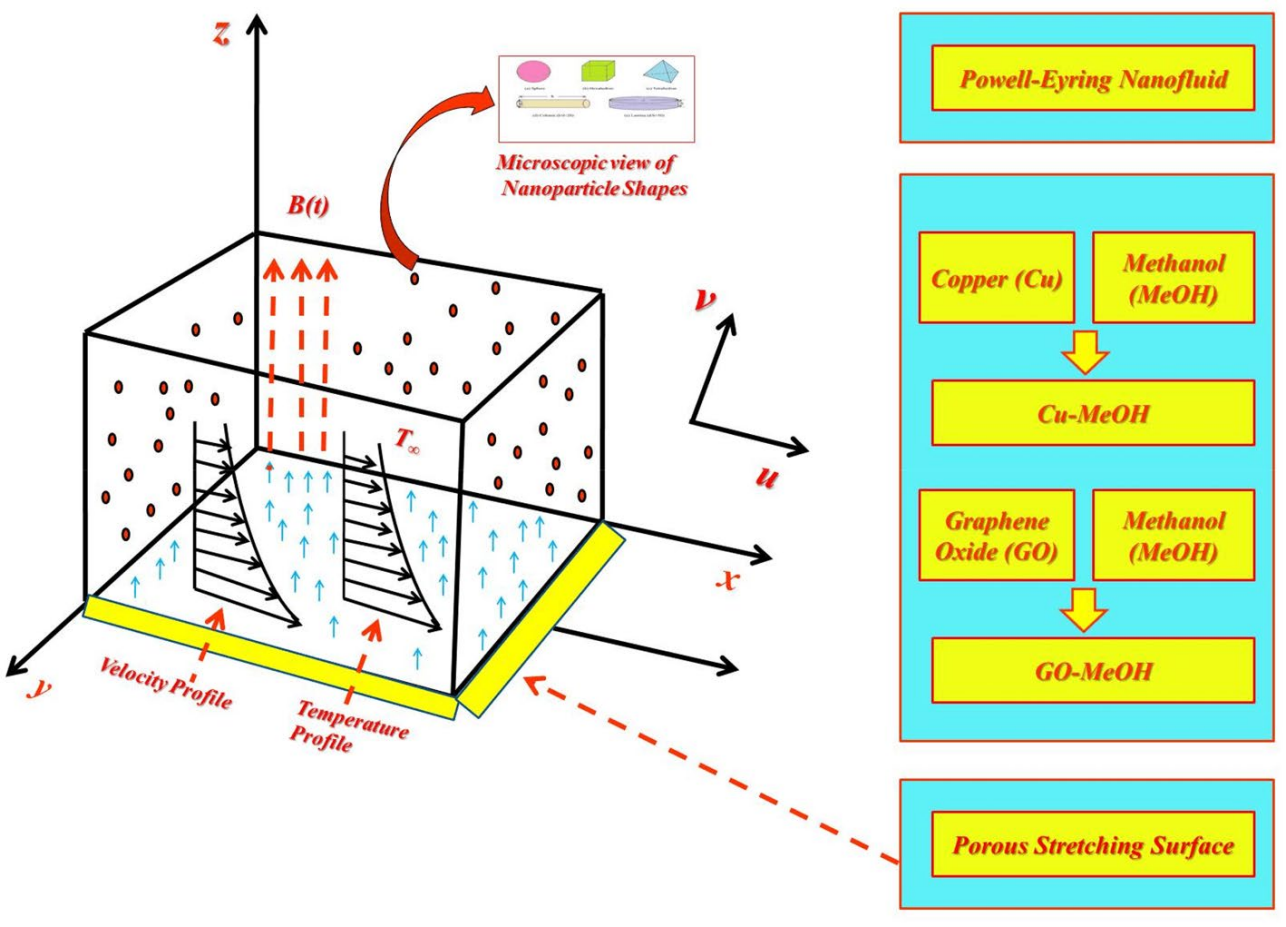
Graphic diagram of nanofluid movement.

2-dimensional transient laminar incompressible electrical conductive Powell-Eyring nanofluid flowing past an expandable surface having non-uniform stretching velocity manifested by

1 $${U}_{w}(x,t)=cx,$$

herein $c$ is a preliminary extending rate. The partial slip, as well as convective conditions, are considered at the boundary. A magnetic field ${B}_{0}$ is utilized in a perpendicular direction to the fluid flow, and an induced magnetic field is neglected in comparison to $B$. The expressions ${T}_{w}(x,t)={T}_{\infty }+cx$ as well as ${T}_{\infty }$ indicates wall and ambient temperature respectively. For convenience, the sheet has to be fixed at $x=0$ and is stretching in the positive $x$-direction. Moreover, the sheet is considered slippery and is subjected to a temperature gradient. Powell-Eyring nanofluid behaves like shear thickening and is assumed optically thick. The stress tensor expression for the case of Power-Eyring fluid is specified by (see, for example, Powell and Eyring^29^)

2 $${\tau }_{ij}={\mu }_{nf}\left(\frac{\partial {u}_{i}}{\partial {x}_{j}}\right)+\frac{1}{\stackrel{\sim }{\beta }}{\mathrm{sinh}}^{-1}\left(\frac{1}{{\varsigma }^{*}}\frac{\partial {u}_{i}}{\partial {x}_{j}}\right),$$

where ${\mu }_{nf}$ indicates dynamic viscosity, $\stackrel{\sim }{\beta }$ and ${\varsigma }^{*}$ for material constants. The inside geometry of the physical model is illustrated in Fig. 1.

The controlling modeled formulas (Kumar and Srinivas et al.^47^) are given by

3 $$\frac{\partial u}{\partial x}+\frac{\partial v}{\partial y}=0,$$

4 $$u\frac{\partial u}{\partial x}+v\frac{\partial u}{\partial y}=\left({\nu }_{nf}+\frac{1}{{\rho }_{nf}\stackrel{\sim }{\beta }{\varsigma }^{*}}\right)\frac{{\partial }^{2}u}{\partial {y}^{2}}-\frac{1}{2\stackrel{\sim }{\beta }{\varsigma }^{{*}^{3}}{\rho }_{nf}}{\left(\frac{\partial u}{\partial y}\right)}^{2}\frac{{\partial }^{2}u}{\partial {y}^{2}}-\frac{{\mu }_{nf}}{{\rho }_{nf}k}u-\frac{{\sigma }_{nf}{B}^{2}u}{{\rho }_{nf}},$$

5 $$u\frac{\partial T}{\partial x}+v\frac{\partial T}{\partial y}=\frac{{k}_{nf}}{(\rho {C}_{p}{)}_{nf}}\left(\frac{{\partial }^{2}T}{\partial {y}^{2}}\right)-\frac{1}{{\left(\rho {C}_{p}\right)}_{nf}}\left(\frac{\partial {q}_{r}}{\partial y}\right)+\frac{{\mu }_{nf}}{{\left(\rho {C}_{p}\right)}_{nf}}{\left(\frac{\partial u}{\partial y}\right)}^{2}+\frac{1}{{\left(\rho {C}_{p}\right)}_{nf}}Q\left(T-{T}_{\infty }\right)+\frac{{\sigma }_{nf}{B}^{2}{u}^{2}}{(\rho {C}_{p}{)}_{nf}}.$$

The BCs are bestowed by (for instance^48^)

6 $$u(x,0)={U}_{w}+{\mu }_{nf}\left(\frac{\partial u}{\partial y}\right), \quad v(x,0)={V}_{w}, \quad -{k}_{0}\left(\frac{\partial T}{\partial y}\right)={h}_{f}({T}_{w}-T),$$

7 $$u\to 0, T\to {T}_{\infty } \quad as \quad y\to \infty .$$

Here, $u$ and $v$ depicts velocities along $x$ as well as $y$ axis, $t$ is the time, $T$ is a temperature of the fluid, ${\mu }_{nf}$ is the dynamical viscidness of the nanofluid, ${\rho }_{nf}$ is the intensity, ${\sigma }_{nf}$ is the electrically conducting. ${q}_{r}$ is the radiative heat flux, $({C}_{p}{)}_{nf}$ and ${\kappa }_{nf}$ are the specific heat capacitance and the thermal conductance, correspondingly. ${V}_{w}$ signifies the penetrability of the expanding sheet. The penetrability of nanofluid is signified by $k$. The expressions regarding thermal conduction and heat transfer coefficient are delineated as ${k}_{0}$ and ${h}_{f}$. Table 1 represents physical properties^49–51^ for the case of Powell-Eyring fluid.

**Table 1:** Thermo-physical characteristics formulas.

Properties	Nanofluid
Dynamic viscosity	${\mu }_{nf}={\mu }_{f}{\left(1-\phi \right)}^{-2.5}$
Density	${\rho }_{nf}=\left(1-\phi \right){\rho }_{f}+\phi {\rho }_{s}$
Heat capacity	$({\rho {C}_{p})}_{nf}=\left(1-\phi \right){(\rho {C}_{p})}_{f}+\phi {(\rho {C}_{p})}_{s}$
Thermal conductivity	$\frac{{k}_{nf}}{{k}_{f}}=\frac{{k}_{s}+\left(m-1\right){k}_{f}-(m-1)\phi ({k}_{f}-{k}_{s})}{{k}_{s}+\left(m-1\right){k}_{f}+\phi ({k}_{f}-{k}_{s})}$
Electrical conductivity	$$\frac{{\sigma_{nf} }}{{\sigma_{f} }} = \left[ {1 + \frac{{3\left( {{\raise0.7ex\hbox{${\sigma_{s} }$} \!\mathord{\left/ {\vphantom {{\sigma_{s} } {\sigma_{f} }}}\right.\kern-\nulldelimiterspace} \!\lower0.7ex\hbox{${\sigma_{f} }$}} - 1} \right)\phi }}{{\left( {{\raise0.7ex\hbox{${\sigma_{s} }$} \!\mathord{\left/ {\vphantom {{\sigma_{s} } {\sigma_{f} }}}\right.\kern-\nulldelimiterspace} \!\lower0.7ex\hbox{${\sigma_{f} }$}} + 2} \right) - \left( {{\raise0.7ex\hbox{${\sigma_{s} }$} \!\mathord{\left/ {\vphantom {{\sigma_{s} } {\sigma_{f} }}}\right.\kern-\nulldelimiterspace} \!\lower0.7ex\hbox{${\sigma_{f} }$}} - 1} \right)\phi }}} \right]$$

Based on Table 1, $\phi$ indicates the nanoparticle volume fraction. Symbols ${\mu }_{f}$, ${\rho }_{f}$ and $({C}_{p}{)}_{f}$, ${\kappa }_{f}$ and ${\sigma }_{f}$ are dynamical viscidness, intensity, specific heat capacitance, the thermally and electrically conductive of the basefluid. ${\rho }_{s}$, $({C}_{p}{)}_{s}$, ${\kappa }_{s}$ and ${\sigma }_{s}$ are the density, specific heat capacity, the thermal and electrically conducting of the nano-solid particles. Table 2 presents empirical shape factor values in the case of distinguished particle shapes (see for example^52, 53^).

**Table 2:** Empirical form factor values for various particle forms.

Nanoparticles type	Sphere	Hexahedron	Tetrahedron	Column	Lamina
Shape					
$m$	3	3.7221	4.0613	6.3698	16.1576

Roseland expression in terms of heat flux (Brewster^54^) is given by

8 $$\frac{\partial {q}_{r}}{\partial y}=-\frac{{2}^{4}{T}_{\infty }^{3}\sigma }{3{k}^{*}}\frac{{\partial }^{2}T}{\partial {y}^{2}}.$$

where $\sigma$ and ${k}^{*}$ points out Stefan/Boltzmann constant and moreover ${k}^{*}$ is the absorption coefficient.

## Solution technique

To obtain the solution of constitutive system (3)–(5) along with BCs (7)–(8), stream functions $\psi$ and $\theta$ are assumed as

9 $$u=\frac{\partial \psi }{\partial y},\quad v=-\frac{\partial \psi }{\partial x}.$$

Similarity variables are defined as

10 $$\chi (x,y)=\sqrt{\frac{c}{{\nu }_{f}}}y,\quad \psi (x,y)=\sqrt{{\nu }_{f}c}xf(\chi ),\quad \theta (\chi )=\frac{T-{T}_{\infty }}{{T}_{w}-{T}_{\infty }}.$$

Utilizing (9)–(10) into (3)–(7) to obtain dimensionless ODEs mentioned underneath.

11 $$\left(\frac{1}{{\phi }_{1}{\phi }_{2}}+\frac{\omega }{{\phi }_{1}}\right)f^{\prime\prime\prime}+ff^{\prime\prime}-{f^{\prime}}^{2}-\frac{\omega\Delta }{{\phi }_{2}}{f^{\prime\prime}}^{2}f^{\prime\prime\prime}-\frac{1}{{\phi }_{1}{\phi }_{2}}Kf^{\prime}-\frac{{\phi }_{4}}{{\phi }_{2}}Mf^{\prime}=0,$$

12 $$\theta ^{\prime\prime}\left(1+\frac{1}{{\phi }_{5}}PrNr\right)+Pr\frac{{\phi }_{3}}{{\phi }_{5}}\left[f\theta ^{\prime}-f^{\prime}\theta +\theta \frac{Q}{{\phi }_{3}}+\frac{Ec}{{\phi }_{1}{\phi }_{3}}{f^{\prime\prime}}^{2}+\frac{{\phi }_{4}}{{\phi }_{3}}MEc{f^{\prime}}^{2}\right]=0,$$

13 $$f(0)=S,\quad f^{\prime}(0)=1+\frac{\Lambda }{{\phi }_{1}}f^{\prime\prime}(0),\quad \theta ^{\prime}(0)=-Bi(1-\theta (0)),$$

14 $$f^{\prime}(\chi )\to 0, \quad\theta (\chi )\to 0, \quad as \quad\chi \to \infty ,$$

where

15 $${\phi }_{1}={\left(1-\phi \right)}^{2.5}, \quad{\phi }_{2}=\left(1-\phi +\phi \frac{{\rho }_{s}}{{\rho }_{f}}\right), \quad {\phi }_{3}=\left(1-\phi +\phi \frac{(\rho {C}_{p}{)}_{s}}{(\rho {C}_{p}{)}_{f}}\right),$$

16 $${\phi }_{4}=\left(1+\frac{3(\frac{{\sigma }_{s}}{{\sigma }_{f}}-1)\phi }{(\frac{{\sigma }_{s}}{{\sigma }_{f}}+2)-(\frac{{\sigma }_{s}}{{\sigma }_{f}}-1)\phi }\right), \quad {\phi }_{5}=\left(\frac{({k}_{s}+(m-1){k}_{f})-(m-1)\phi ({k}_{f}-{k}_{s})}{({k}_{s}+(m-1){k}_{f})+\phi ({k}_{f}-{k}_{s})}\right).$$

Prime refers to the differentiation with regards to $\chi$ in these formulas, $\omega =\frac{1}{{\mu }_{f}\stackrel{\sim }{\beta }{\varsigma }^{*}}$ and $\Delta =\frac{{{U}_{w}}^{2}}{2{\varsigma }^{{*}^{2}}{\nu }_{f}x}$ are the material parameters respectively, $M=\frac{{\sigma }_{f}{B}_{0}^{2}}{c{\rho }_{f}}$ and $K=\frac{{\nu }_{f}}{ck}$ are the magnetic and porous media parameters respectively, $Pr=\frac{{\nu }_{f}}{{\alpha }_{f}}$ is the Prandtl number, ${\alpha }_{f}=\frac{{\kappa }_{f}}{(\rho {C}_{p}{)}_{f}}$ is the thermal diffusion parameter, $Nr=\frac{16}{3}\frac{{\sigma }^{*}{T}_{\infty }^{3}}{{\kappa }^{*}{\nu }_{f}(\rho {C}_{p}{)}_{f}}$ is the thermal radiative factor, $Q=\frac{{Q}_{0}}{(\rho {C}_{p}{)}_{f}c}$ is the heat generation, $S=-{V}_{w}\sqrt{\frac{1}{{\nu }_{f} c}}$ is the mass transfer parameter, $\Lambda =\sqrt{\frac{c}{{\nu }_{f}}}{\mu }_{f}$ is the slippy factor and expression $Ec=\frac{{U}_{w}^{2}}{({C}_{p}{)}_{f}({T}_{w}-{T}_{\infty })}$ and expressions $Bi=\frac{{h}_{f}}{{k}_{0}}\sqrt{\frac{{\nu }_{f}}{c}}$ indicates Eckert as well as Biot number.

## Classical Keller-Box numerical technique

**Figure 2:**
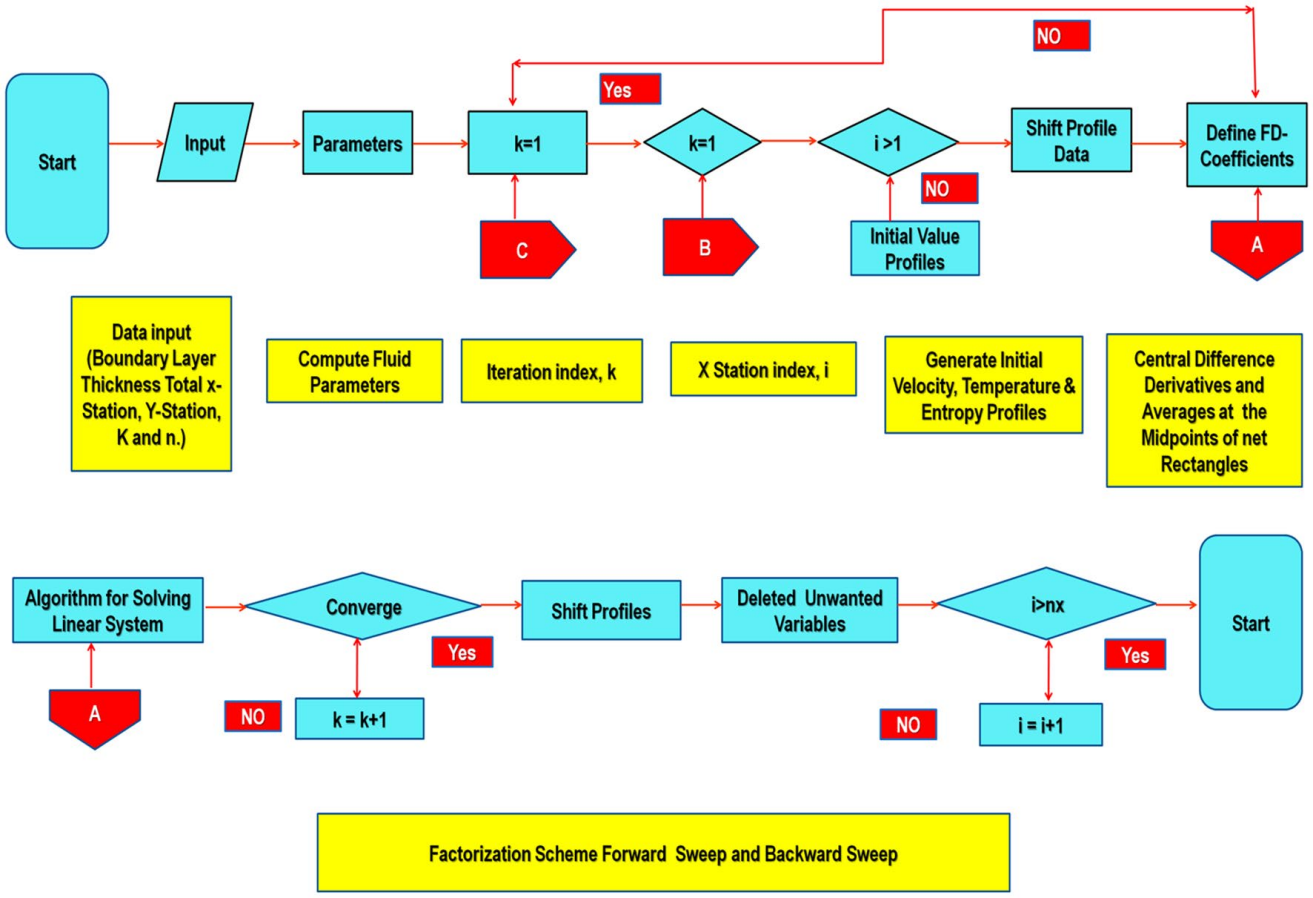
Keller-box method flowchart.

Keller-box method (KBM)^55^ is utilized to obtain the numerical solution of modeled equations. This method generally provides fast convergence in contrast to other nonlinear numerical schemes. This scheme provides convergent up to second-order and inherently stable as well. This method assures Von Neumann's stability test in terms of stability analysis. This test sets the criterion for the convergence of the numerical solution to PDEs’ real solution using the numerical solution’s consistency and stability. The solutions of Eqs. (11)–(12) along with, (13)–(14), are achieved by KBM. The flow chart mechanism of Keller box method is explained below. (see Fig. 2):

### Stage 1: renovation of ODEs

The ODEs (11)–(14) are stepped down into five first-order ODEs mentioned below

17 $${z}_{1}={f}^{^{\prime}},$$

18 $${z}_{2}={u}^{^{\prime}},$$

19 $${z}_{3}={\theta }^{^{\prime}},$$

20 $$\left(\frac{1}{{\phi }_{1}{\phi }_{2}}+\frac{\omega }{{\phi }_{1}}\right){z}_{2}^{^{\prime}}+f{z}_{2}-{z}_{1}^{2}-\frac{\omega\Delta }{{\phi }_{2}}{z}_{2}^{2}{z}_{2}^{^{\prime}}-\frac{1}{{\phi }_{1}{\phi }_{2}}K{z}_{1}-\frac{{\phi }_{4}}{{\phi }_{2}}M{z}_{1}=0,$$

21 $${z}_{3}^{^{\prime}}\left(1+\frac{1}{{\phi }_{5}}PrNr\right)+Pr\frac{{\phi }_{3}}{{\phi }_{5}}\left[f{z}_{3}-{z}_{1}\theta +\theta \frac{Q}{{\phi }_{3}}+\frac{Ec}{{\phi }_{1}{\phi }_{3}}{z}_{2}^{2}+\frac{{\phi }_{4}}{{\phi }_{3}}MEc{z}_{1}^{2}\right]=0,$$

22 $$f(0)=S,{z}_{1}(0)=1+\frac{\Lambda }{{\phi }_{1}}{z}_{2}(0),{z}_{3}(0)=-Bi(1-\theta (0)),{z}_{1}(\infty )\to 0,\theta (\infty )\to 0.$$

### Stage 2: discretize domain

**Figure 3:**
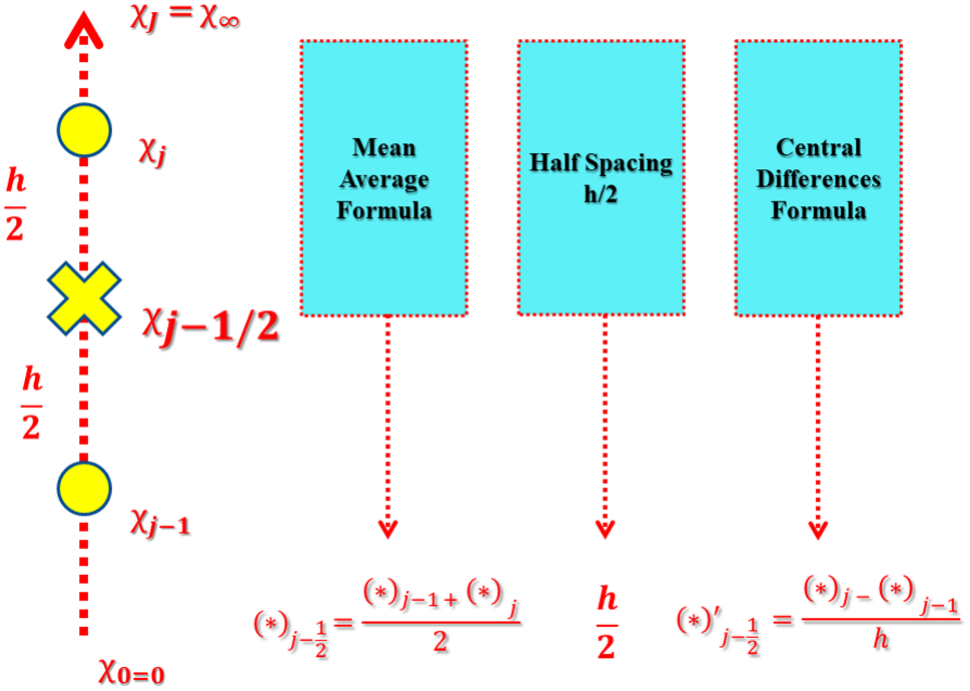
Net rectangle for difference approximations.

The discretization of a domain can be done by dividing the domain of the system into small uniform grids to obtain the approximate solution (see Fig. 3). Generally smaller grid provides high accuracy (6).

$${\chi }_{0}=0, \quad {\chi }_{j}={\chi }_{j-1}+h,\quad j=\mathrm{1,2},3,...,J-1, \quad {\chi }_{J}={\chi }_{\infty }.$$

In this problem, the value of $h$ is fixed to $0.01$. To achieved difference equations the process of central differences has been implemented. Mean averages replace the functions. The ODEs (17)–(22) are transformed into algebraic expressions of nonlinear nature.

23 $$\frac{{{z}_{1}}_{j}+{{z}_{1}}_{j-1}}{2}=\frac{{f}_{j}-{f}_{j-1}}{h},$$

24 $$\frac{{{z}_{2}}_{j}+{{z}_{2}}_{j-1}}{2}=\frac{{{z}_{1}}_{j}-{{z}_{1}}_{j-1}}{h},$$

25 $$\frac{({z}_{3}{)}_{j}+({z}_{3}{)}_{j-1}}{2}=\frac{{\theta }_{j}-{\theta }_{j-1}}{h},$$

26 $$\begin{array}{l}\left(\frac{1}{{\phi }_{1}{\phi }_{2}}+\frac{\omega }{{\phi }_{1}}\right)\left(\frac{({z}_{2}{)}_{j}-({z}_{2}{)}_{j-1}}{h}\right)+\left(\frac{{f}_{j}+{f}_{j-1}}{2}\right)\left(\frac{({z}_{2}{)}_{j}+({z}_{2}{)}_{j-1}}{2}\right)\\ \quad-\,{\left(\frac{({z}_{1}{)}_{j}+({z}_{1}{)}_{j-1}}{2}\right)}^{2}-\frac{\omega\Delta }{{\phi }_{2}}{\left(\frac{({z}_{2}{)}_{j}+({z}_{2}{)}_{j-1}}{2}\right)}^{2}\left(\frac{({z}_{2}{)}_{j}+({z}_{2}{)}_{j-1}}{h}\right)-\frac{{\phi }_{4}}{{\phi }_{2}}M\left(\frac{({z}_{1}{)}_{j}+({z}_{1}{)}_{j-1}}{2}\right)\\ \quad-\,\frac{1}{{\phi }_{1}{\phi }_{2}}K\left(\frac{({z}_{1}{)}_{j}+({z}_{1}{)}_{j-1}}{2}\right)=0,\end{array}$$

27 $$\begin{array}{l}\left(\frac{({z}_{3}{)}_{j}-({z}_{3}{)}_{j-1}}{h}\right)\left(1+\frac{1}{{\phi }_{5}}PrNr\right)+Pr\frac{{\phi }_{3}}{{\phi }_{5}}\left[\left(\frac{{f}_{j}+{f}_{j-1}}{2}\right)\left(\frac{({z}_{3}{)}_{j}+({z}_{3}{)}_{j-1}}{2}\right)\right]\\ \quad +\,Pr\frac{{\phi }_{3}}{{\phi }_{5}}\left[\frac{Q}{{\phi }_{3}}\left(\frac{{\theta }_{j}+{\theta }_{j-1}}{2}\right)-\left(\frac{({z}_{1}{)}_{j}+({z}_{1}{)}_{j-1}}{2}\right)\left(\frac{{\theta }_{j}+{\theta }_{j-1}}{2}\right)+\frac{Ec}{{\phi }_{1}{\phi }_{3}}{\left(\frac{({z}_{2}{)}_{j}+({z}_{2}{)}_{j-1}}{2}\right)}^{2}\right]\\ \quad+\,Pr\frac{{\phi }_{3}}{{\phi }_{5}}\left[\frac{{\phi }_{4}}{{\phi }_{3}}EcM{\left(\frac{({z}_{1}{)}_{j}+({z}_{1}{)}_{j-1}}{2}\right)}^{2}\right]=0.\end{array}$$

### Stage 3: linearized by via Newton’s technique

Newton’s technique has been implemented to linearize the subsequent system of formulas. The $(i+1)th$ iteration in terms of the above equations are denoted by

28 $$({)}_{j}^{(i+1)}=({)}_{j}^{(i)}+\varpi ({)}_{j}^{(i)},$$

The replacement of overhead in formulas (23)–(27) and disregard the quadratic and higher bounds of ${\varpi }_{j}^{i}$, a linear tri-diagonal system is achieved

29 $$\varpi {f}_{j}-\varpi {f}_{j-1}-\frac{1}{2}h(\varpi {{z}_{1}}_{j}+\varpi {{z}_{1}}_{j-1})=({r}_{1}{)}_{j-\frac{1}{2}},$$

30 $$\varpi {{z}_{1}}_{j}-\varpi {{z}_{1}}_{j-1}-\frac{1}{2}h(\varpi {{z}_{2}}_{j}+\varpi {{z}_{2}}_{j-1})=({r}_{2}{)}_{j-\frac{1}{2}},$$

31 $$\varpi {\theta }_{j}-\varpi {\theta }_{j-1}-\frac{1}{2}h(\varpi ({z}_{3}{)}_{j}+\varpi ({z}_{3}{)}_{j-1})=({r}_{3}{)}_{j-\frac{1}{2}},$$

32 $$\begin{array}{l}({a}_{1}{)}_{j}\varpi {f}_{j}+({a}_{2}{)}_{j}\varpi {f}_{j-1}+({a}_{3}{)}_{j}\varpi {{z}_{1}}_{j}+({a}_{4}{)}_{j}\varpi {{z}_{1}}_{j-1}+({a}_{5}{)}_{j}\varpi {{z}_{2}}_{j}+({a}_{6}{)}_{j}\varpi {{z}_{2}}_{j-1}\\ \quad +\,({a}_{7}{)}_{j}\varpi {\theta }_{j}+({a}_{8}{)}_{j}\varpi {\theta }_{j-1}+({a}_{9}{)}_{j}\varpi ({z}_{3}{)}_{j}+({a}_{10}{)}_{j}\varpi ({z}_{3}{)}_{j-1}=({r}_{4}{)}_{j-\frac{1}{2}},\end{array}$$

33 $$\begin{array}{l}({b}_{1}{)}_{j}\varpi {f}_{j}+({b}_{2}{)}_{j}\varpi {f}_{j-1}+({b}_{3}{)}_{j}\varpi {{z}_{1}}_{j}+({b}_{4}{)}_{j}\varpi {{z}_{1}}_{j-1}+({b}_{5}{)}_{j}\varpi {{z}_{2}}_{j}+({b}_{6}{)}_{j}\varpi {{z}_{2}}_{j-1}\\ \quad+\,({b}_{7}{)}_{j}\varpi {\theta }_{j}+({b}_{8}{)}_{j}\varpi {\theta }_{j-1}+({b}_{9}{)}_{j}\varpi ({z}_{3}{)}_{j}+({b}_{10}{)}_{j}\varpi ({z}_{3}{)}_{j-1}=({r}_{5}{)}_{j-\frac{1}{2}}.\end{array}$$

where

34 $$({r}_{1}{)}_{j-\frac{1}{2}}=-{f}_{j}+{f}_{j-1}+\frac{h}{2}({{z}_{1}}_{j}+{{z}_{1}}_{j-1}),$$

35 $$({r}_{2}{)}_{j-\frac{1}{2}}=-{{z}_{1}}_{j}+{{z}_{1}}_{j-1}+\frac{h}{2}({{z}_{2}}_{j}+{{z}_{2}}_{j-1}),$$

36 $$({r}_{3}{)}_{j-\frac{1}{2}}=-{\theta }_{j}+{\theta }_{j-1}+\frac{h}{2}(({z}_{3}{)}_{j}+({z}_{3}{)}_{j-1}),$$

37 $$\begin{aligned}&({r}_{4}{)}_{j-\frac{1}{2}}=h\left[\left(\frac{1}{{\phi }_{1}{\phi }_{2}}+\frac{\omega }{{\phi }_{1}}\right)\left(\frac{(({z}_{2}{)}_{j}-({z}_{2}{)}_{j-1})}{h}\right)\right]+h\left[\left(\frac{({f}_{j}+{f}_{j-1})(({z}_{2}{)}_{j}+({z}_{2}{)}_{j-1})}{4}\right)\right]\\&\quad-\,h\left[{\left(\frac{({z}_{1}{)}_{j}+({z}_{1}{)}_{j-1}}{2}\right)}^{2}+\frac{\omega\Delta }{{\phi }_{2}}{\left(\frac{({z}_{2}{)}_{j}+({z}_{2}{)}_{j-1}}{2}\right)}^{2}\left(\frac{({z}_{2}{)}_{j}+({z}_{2}{)}_{j-1}}{h}\right)\right]-h\left[\frac{1}{{\phi }_{1}{\phi }_{2}}K\left(\frac{({z}_{1}{)}_{j}+({z}_{1}{)}_{j-1}}{2}\right)\right.\\&\quad\left.+\,\frac{{\phi }_{4}}{{\phi }_{2}}M\left(\frac{({z}_{1}{)}_{j}+({z}_{1}{)}_{j-1}}{2}\right)\right],\end{aligned}$$

38 $$\begin{aligned}&({r}_{5}{)}_{j-\frac{1}{2}}=-h\left[\left(\frac{(({z}_{3}{)}_{j}-({z}_{3}{)}_{j-1})}{h}\right)\left(1+\frac{1}{{\phi }_{5}}PrNr\right)+\frac{{\phi }_{3}Pr}{{\phi }_{5}}\left(\frac{({f}_{j}+{f}_{j-1})(({z}_{3}{)}_{j}+({z}_{3}{)}_{j-1})}{4}\right)\right]\\&\quad+\,h\frac{{\phi }_{3}Pr}{{\phi }_{5}}\left[\left(\frac{(({z}_{3}{)}_{j}+({z}_{3}{)}_{j-1})({{z}_{1}}_{j}+{{z}_{1}}_{j-1})}{4}\right)+\frac{Q}{{\phi }_{3}}\left(\frac{{\theta }_{j}+{\theta }_{j-1}}{2}\right)\right]-h\frac{{\phi }_{3}Pr}{{\phi }_{5}}\left[\frac{Ec}{{\phi }_{1}{\phi }_{3}}{\left(\frac{({z}_{2}{)}_{j}+({z}_{2}{)}_{j-1}}{2}\right)}^{2}\right.\\&\quad\left.+\,\frac{{\phi }_{4}}{{\phi }_{3}}EcM{\left(\frac{({z}_{1}{)}_{j}+({z}_{1}{)}_{j-1}}{2}\right)}^{2}\right].\end{aligned}$$

The boundary constraints become through the similarity procedure

39 $$\varpi {f}_{0}=0,\varpi ({z}_{1}{)}_{0}=0,\varpi ({z}_{3}{)}_{0}=0,\varpi ({z}_{1}{)}_{J}=0,\varpi {\theta }_{J}=0,$$

### Stage 4: the block-tridiagonal matrix

The above formulas (29)–(33) have a tridiagonal block structure. In a matrix–vector, we write the system accordingly,

For $$j=1;$$

40 $$\varpi {f}_{1}-\varpi {f}_{0}-\frac{1}{2}h(\varpi ({z}_{1}{)}_{1}+\varpi ({z}_{1}{)}_{0})=({r}_{1}{)}_{1-\frac{1}{2}},$$

41 $$\varpi ({z}_{1}{)}_{1}-\varpi ({z}_{1}{)}_{0}-\frac{1}{2}h(\varpi ({z}_{2}{)}_{1}+\varpi ({z}_{2}{)}_{0})=({r}_{2}{)}_{1-\frac{1}{2}},$$

42 $$\varpi {\theta }_{1}-\varpi {\theta }_{0}-\frac{1}{2}h(\varpi ({z}_{3}{)}_{1}+\varpi ({z}_{3}{)}_{0})=({r}_{3}{)}_{1-\frac{1}{2}},$$

43 $$\begin{array}{l}({a}_{1}{)}_{1}\varpi {f}_{1}+({a}_{2}{)}_{1}\varpi {f}_{0}+({a}_{3}{)}_{1}\varpi {{z}_{1}}_{1}+({a}_{4}{)}_{1}\varpi {{z}_{1}}_{0}+({a}_{5}{)}_{1}\varpi {{z}_{2}}_{1}+({a}_{6}{)}_{1}\varpi {{z}_{2}}_{0}\\ \quad+\,({a}_{7}{)}_{1}\varpi {\theta }_{j}+({a}_{8}{)}_{1}\varpi {\theta }_{0}+({a}_{9}{)}_{1}\varpi ({z}_{3}{)}_{1}+({a}_{10}{)}_{1}\varpi ({z}_{3}{)}_{0}=({r}_{4}{)}_{1-\frac{1}{2}},\end{array}$$

44 $$\begin{array}{l}({b}_{1}{)}_{1}\varpi {f}_{1}+({b}_{2}{)}_{1}\varpi {f}_{0}+({b}_{3}{)}_{1}\varpi {{z}_{1}}_{1}+({b}_{4}{)}_{1}\varpi {{z}_{1}}_{0}+({b}_{5}{)}_{1}\varpi {{z}_{2}}_{1}+({b}_{6}{)}_{1}\varpi {{z}_{2}}_{0}\\\quad +\,({b}_{7}{)}_{1}\varpi {\theta }_{1}+({b}_{8}{)}_{1}\varpi {\theta }_{0}+({b}_{9}{)}_{1}\varpi ({z}_{3}{)}_{1}+({b}_{10}{)}_{1}\varpi ({z}_{3}{)}_{0}=({r}_{5}{)}_{1-\frac{1}{2}}.\end{array}$$

In matrix formula,

45 $$\left[\begin{array}{lllll}0& 0& 1& 0& 0\\ -h/2& 0& 0& -h/2& 0\\ 0& -h/2& 0& 0& -h/2\\ ({a}_{2}{)}_{1}& ({a}_{10}{)}_{1}& ({a}_{3}{)}_{1}& ({a}_{1}{)}_{1}& ({a}_{9}{)}_{1}\\ ({b}_{2}{)}_{1}& ({b}_{10}{)}_{1}& ({b}_{3}{)}_{1}& ({b}_{1}{)}_{1}& ({b}_{9}{)}_{1}\end{array}\right]\left[\begin{array}{l}\varpi ({z}_{2}{)}_{0}\\ \varpi (\theta {)}_{0}\\ \varpi (f{)}_{1}\\ \varpi ({z}_{2}{)}_{1}\\ \varpi ({z}_{3}{)}_{1}\end{array}\right]+\left[\begin{array}{lllll}-h/2& 0& 0& 0& 0\\ 1& 0& 0& 0& 0\\ 0& 1& 0& 0& 0\\ ({a}_{5}{)}_{1}& ({a}_{7}{)}_{1}& 0& 0& 0\\ ({b}_{5}{)}_{1}& ({b}_{7}{)}_{1}& 0& 0& 0\end{array}\right]\left[\begin{array}{l}\varpi ({z}_{1}{)}_{1}\\ \varpi (\theta {)}_{1}\\ \varpi (f{)}_{2}\\ \varpi ({z}_{2}{)}_{2}\\ \varpi ({z}_{3}{)}_{2}\end{array}\right]=\left[\begin{array}{l}({r}_{1}{)}_\frac{1}{2}\\ ({r}_{2}{)}_\frac{1}{2}\\ ({r}_{3}{)}_\frac{1}{2}\\ ({r}_{4}{)}_\frac{1}{2}\\ ({r}_{5}{)}_\frac{1}{2}\end{array}\right].$$

That is

46 $$[{A}_{1}][{\varpi }_{1}]+[{C}_{1}][{\varpi }_{2}]=[{r}_{1}].$$

For $$j=2;$$

47 $$\varpi {f}_{2}-\varpi {f}_{1}-\frac{1}{2}h(\varpi ({z}_{1}{)}_{2}+\varpi ({z}_{1}{)}_{1})=({r}_{1}{)}_{1-\frac{1}{2}},$$

48 $$\varpi ({z}_{1}{)}_{2}-\varpi ({z}_{1}{)}_{1}-\frac{1}{2}h(\varpi ({z}_{2}{)}_{2}+\varpi ({z}_{2}{)}_{1})=({r}_{2}{)}_{1-\frac{1}{2}},$$

49 $$\varpi {\theta }_{1}-\varpi {\theta }_{0}-\frac{1}{2}h(\varpi ({z}_{3}{)}_{2}+\varpi ({z}_{3}{)}_{1})=({r}_{3}{)}_{1-\frac{1}{2}},$$

50 $$\begin{array}{l}({a}_{1}{)}_{2}\varpi {f}_{2}+({a}_{2}{)}_{2}\varpi {f}_{1}+({a}_{3}{)}_{2}\varpi {{z}_{1}}_{2}+({a}_{4}{)}_{2}\varpi {{z}_{1}}_{1}+({a}_{5}{)}_{2}\varpi {{z}_{2}}_{2}+({a}_{6}{)}_{2}\varpi {{z}_{2}}_{1}\\ \quad+\,({a}_{7}{)}_{2}\varpi {\theta }_{2}+({a}_{8}{)}_{2}\varpi {\theta }_{1}+({a}_{9}{)}_{2}\varpi ({z}_{3}{)}_{2}+({a}_{10}{)}_{2}\varpi ({z}_{3}{)}_{1}=({r}_{4}{)}_{2-\frac{1}{2}},\end{array}$$

51 $$\begin{array}{l}({b}_{1}{)}_{2}\varpi {f}_{2}+({b}_{2}{)}_{2}\varpi {f}_{1}+({b}_{3}{)}_{2}\varpi {{z}_{1}}_{2}+({b}_{4}{)}_{2}\varpi {{z}_{1}}_{1}+({b}_{5}{)}_{2}\varpi {{z}_{2}}_{2}+({b}_{6}{)}_{2}\varpi {{z}_{2}}_{1}\\ \quad+\,({b}_{7}{)}_{2}\varpi {\theta }_{2}+({b}_{8}{)}_{2}\varpi {\theta }_{1}+({b}_{9}{)}_{2}\varpi ({z}_{3}{)}_{2}+({b}_{10}{)}_{2}\varpi ({z}_{3}{)}_{1}=({r}_{5}{)}_{2-\frac{1}{2}}.\end{array}$$

In matrix formula,

52 $$\begin{aligned}&\left[\begin{array}{lllll}0& 0& -1& 0& 0\\ 0& 0& 0& -\frac{h}{2}& 0\\ 0& 0& 0& 0& -\frac{h}{2}\\ 0& 0& ({a}_{4}{)}_{2}& ({a}_{2}{)}_{2}& ({a}_{10}{)}_{2}\\ 0& 0& ({b}_{4}{)}_{2}& ({b}_{2}{)}_{2}& ({b}_{10}{)}_{2}\end{array}\right]\left[\begin{array}{l}\varpi ({z}_{2}{)}_{0}\\ \varpi (\theta {)}_{0}\\ \varpi (f{)}_{1}\\ \varpi ({z}_{2}{)}_{1}\\ \varpi ({z}_{3}{)}_{1}\end{array}\right]\\&\quad\quad+\left[\begin{array}{lllll}-\frac{h}{2}& 0& 1& 0& 0\\ -1& 0& 0& -\frac{h}{2}& 0\\ 0& -1& 0& 0& -\frac{h}{2}\\ ({a}_{6}{)}_{2}& ({a}_{8}{)}_{2}& ({a}_{3}{)}_{2}& ({a}_{1}{)}_{2}& ({a}_{9}{)}_{2}\\ ({b}_{6}{)}_{2}& ({b}_{8}{)}_{2}& ({b}_{3}{)}_{2}& ({b}_{1}{)}_{2}& ({b}_{9}{)}_{2}\end{array}\right]\left[\begin{array}{l}\varpi ({z}_{1}{)}_{1}\\ \varpi (\theta {)}_{1}\\ \varpi (f{)}_{2}\\ \varpi ({z}_{2}{)}_{2}\\ \varpi ({z}_{3}{)}_{2}\end{array}\right]\left[\begin{array}{lllll}-\frac{h}{2}& 0& 1& 0& 0\\ 1& 0& 0& -\frac{h}{2}& 0\\ 0& 1& 0& 0& -\frac{h}{2}\\ ({a}_{5}{)}_{2}& ({a}_{7}{)}_{2}& 0& 0& 0\\ ({b}_{5}{)}_{2}& ({b}_{7}{)}_{2}& 0& 0& 0\end{array}\right]\left[\begin{array}{l}\varpi ({z}_{1}{)}_{1}\\ \varpi (\theta {)}_{1}\\ \varpi (f{)}_{2}\\ \varpi ({z}_{2}{)}_{2}\\ \varpi ({z}_{3}{)}_{2}\end{array}\right]\\&\quad=\left[\begin{array}{l}({r}_{1}{)}_\frac{3}{2}\\ ({r}_{2}{)}_\frac{3}{2}\\ ({r}_{3}{)}_\frac{3}{2}\\ ({r}_{4}{)}_\frac{3}{2}\\ ({r}_{5}{)}_\frac{3}{2}\end{array}\right].\end{aligned}$$

That is

53 $$[{B}_{2}][{\varpi }_{1}]+[{A}_{2}][{\varpi }_{2}]+[{C}_{2}][{\varpi }_{3}]=[{r}_{2}].$$

For $$j=J-1;$$

54 $$\varpi {f}_{J-1}-\varpi {f}_{J-2}-\frac{1}{2}h(\varpi ({z}_{1}{)}_{J-1}+\varpi {{z}_{1}}_{J-2})=({r}_{1}{)}_{J-1-\frac{1}{2}},$$

55 $$\varpi ({z}_{1}{)}_{J-1}-\varpi ({z}_{1}{)}_{J-2}-\frac{1}{2}h(\varpi ({z}_{2}{)}_{J-1}+\varpi ({z}_{2}{)}_{J-2})=({r}_{2}{)}_{J-1-\frac{1}{2}},$$

56 $$\varpi {\theta }_{J-1}-\varpi {\theta }_{J-2}-\frac{1}{2}h(\varpi ({z}_{3}{)}_{J-1}+\varpi ({z}_{3}{)}_{J-2})=({r}_{3}{)}_{J-1-\frac{1}{2}},$$

57 $$\begin{array}{l}({a}_{1}{)}_{J-1}\varpi {f}_{J-1}+({a}_{2}{)}_{J-1}\varpi {f}_{J-2}+({a}_{3}{)}_{J-1}\varpi {{z}_{1}}_{J-1}+({a}_{4}{)}_{J-1}\varpi {{z}_{1}}_{J-2}+({a}_{5}{)}_{J-1}\varpi {{z}_{2}}_{j}\\ \quad\quad+\,({a}_{6}{)}_{J-1}\varpi {{z}_{2}}_{J-2}+({a}_{7}{)}_{J-1}\varpi {\theta }_{J-1}+({a}_{8}{)}_{J-1}\varpi {\theta }_{J-2}+({a}_{9}{)}_{J-1}\varpi ({z}_{3}{)}_{J-1}+({a}_{10}{)}_{J-1}\varpi ({z}_{3}{)}_{J-2}\\\quad =({r}_{4}{)}_{J-1-\frac{1}{2}},\end{array}$$

58 $$\begin{array}{l}({b}_{1}{)}_{J-1}\varpi {f}_{J-1}+({b}_{2}{)}_{J-1}\varpi {f}_{J-2}+({b}_{3}{)}_{J-1}\varpi {{z}_{1}}_{J-1}+({b}_{4}{)}_{J-1}\varpi {{z}_{1}}_{J-2}+({b}_{5}{)}_{J-1}\varpi {{z}_{2}}_{J-1}\\\quad\quad +\,({b}_{6}{)}_{J-1}\varpi {{z}_{2}}_{J-2}+({b}_{7}{)}_{J-1}\varpi {\theta }_{J-1}+({b}_{8}{)}_{J-1}\varpi {\theta }_{J-2}+({b}_{9}{)}_{J-1}\varpi ({z}_{3}{)}_{J-1}+({b}_{10}{)}_{J-1}\varpi ({z}_{3}{)}_{J-2}\\\quad =({r}_{5}{)}_{J-1-\frac{1}{2}}.\end{array}$$

In matrix formula,

59 $$\begin{aligned}&\left[\begin{array}{lllll}0& 0& -1& 0& 0\\ 0& 0& 0& -h/2& 0\\ 0& 0& 0& 0& -h/2\\ 0& 0& ({a}_{4}{)}_{J-2}& ({a}_{2}{)}_{J-2}& ({a}_{10}{)}_{J/2}\\ 0& 0& ({b}_{4}{)}_{J-2}& ({b}_{2}{)}_{J-2}& ({b}_{10}{)}_{J-2}\end{array}\right]\left[\begin{array}{l}\varpi ({z}_{2}{)}_{J-3}\\ \varpi (\theta {)}_{J-3}\\ \varpi (f{)}_{J-2}\\ \varpi ({z}_{2}{)}_{J-2}\\ \varpi ({z}_{3}{)}_{J-2}\end{array}\right]\\&\quad+\,\left[\begin{array}{lllll}-h/2& 0& 1& 0& 0\\ -1& 0& 0& -h/2& 0\\ 0& -1& 0& 0& -h/2\\ ({a}_{6}{)}_{J-2}& ({a}_{8}{)}_{J-2}& ({a}_{3}{)}_{J-2}& ({a}_{1}{)}_{J-2}& ({a}_{9}{)}_{J-2}\\ ({b}_{6}{)}_{J-2}& ({b}_{8}{)}_{J-2}& ({b}_{3}{)}_{J-2}& ({b}_{1}{)}_{J-2}& ({b}_{9}{)}_{J-2}\end{array}\right]\left[\begin{array}{l}\varpi ({z}_{2}{)}_{J-2}\\ \varpi (\theta {)}_{J-2}\\ \varpi (f{)}_{J-1}\\ \varpi ({z}_{2}{)}_{J-1}\\ \varpi ({z}_{3}{)}_{J-1}\end{array}\right]\\&\quad+\,\left[\begin{array}{lllll}-h/2& 0& 0& 0& 0\\ 1& 0& 0& 0& 0\\ 0& 1& 0& 0& 0\\ ({a}_{5}{)}_{J-2}& ({a}_{9}{)}_{J-2}& 0& 0& 0\\ ({b}_{5}{)}_{J-2}& ({b}_{9}{)}_{J-2}& 0& 0& 0\end{array}\right]\left[\begin{array}{l}\varpi ({z}_{1}{)}_{J-1}\\ \varpi (\theta {)}_{J-1}\\ \varpi (f{)}_{J}\\ \varpi ({z}_{2}{)}_{J}\\ \varpi ({z}_{3}{)}_{J}\end{array}\right]=\left[\begin{array}{l}({r}_{1}{)}_{(J-1)-\frac{1}{2}}\\ ({r}_{2}{)}_{(J-1)-\frac{1}{2}}\\ ({r}_{3}{)}_{(J-1)-\frac{1}{2}}\\ ({r}_{4}{)}_{(J-1)-\frac{1}{2}}\\ ({r}_{5}{)}_{(J-1)-\frac{1}{2}}\end{array}\right].\end{aligned}$$

That is

60 $$[{B}_{J-1}][{\varpi }_{J-2}]+[{A}_{J-1}][{\varpi }_{J-1}]+[{C}_{J-1}][{\varpi }_{J}]=[{r}_{J-1}].$$

For $$j=J;$$

61 $$\varpi {f}_{J}-\varpi {f}_{J-1}-\frac{1}{2}h(\varpi ({z}_{1}{)}_{J}+\varpi ({z}_{1}{)}_{J-1})=({r}_{1}{)}_{J-\frac{1}{2}},$$

62 $$\varpi ({z}_{1}{)}_{J}-\varpi ({z}_{1}{)}_{J-1}-\frac{1}{2}h(\varpi ({z}_{2}{)}_{J}+\varpi ({z}_{2}{)}_{J-1})=({r}_{2}{)}_{J-\frac{1}{2}},$$

63 $$\varpi {\theta }_{J}-\varpi {\theta }_{J-1}-\frac{1}{2}h(\varpi ({z}_{3}{)}_{J}+\varpi ({z}_{3}{)}_{J-1})=({r}_{3}{)}_{J-\frac{1}{2}},$$

64 $$\begin{array}{l}({a}_{1}{)}_{J}\varpi {f}_{J}+({a}_{2}{)}_{J}\varpi {f}_{J-1}+({a}_{3}{)}_{J}\varpi {{z}_{1}}_{J}+({a}_{4}{)}_{J}\varpi {{z}_{1}}_{J-1}+({a}_{5}{)}_{J}\varpi {{z}_{2}}_{J}+({a}_{6}{)}_{J}\varpi {{z}_{2}}_{J-1}\\ \quad +\,({a}_{7}{)}_{J}\varpi {\theta }_{J}+({a}_{8}{)}_{J}\varpi {\theta }_{J-1}+({a}_{9}{)}_{J}\varpi ({z}_{3}{)}_{J}+({a}_{10}{)}_{J}\varpi ({z}_{3}{)}_{J-1}=({r}_{4}{)}_{J-\frac{1}{2}},\end{array}$$

65 $$\begin{array}{l}({b}_{1}{)}_{J}\varpi {f}_{J}+({b}_{2}{)}_{J}\varpi {f}_{J-1}+({b}_{3}{)}_{J}\varpi {{z}_{1}}_{J}+({b}_{4}{)}_{J}\varpi {{z}_{1}}_{J-1}+({b}_{5}{)}_{J}\varpi {{z}_{2}}_{J}+({b}_{6}{)}_{J}\varpi {{z}_{2}}_{J-1}\\ \quad+\,({b}_{7}{)}_{J}\varpi {\theta }_{J}+({b}_{8}{)}_{J}\varpi {\theta }_{J-1}+({b}_{9}{)}_{J}\varpi ({z}_{3}{)}_{J}+({b}_{10}{)}_{J}\varpi ({z}_{3}{)}_{J-1}=({r}_{5}{)}_{J-\frac{1}{2}}.\end{array}$$

In matrix formula,

66 $$\begin{aligned}&\left[\begin{array}{lllll}-h/2& 0& 1& 0& 0\\ -1& 0& 0& -h/2& 0\\ 0& -1& 0& 0& -h/2\\ ({a}_{6}{)}_{1}& ({a}_{8}{)}_{1}& ({a}_{3}{)}_{1}& ({a}_{1}{)}_{1}& ({a}_{9}{)}_{1}\\ ({b}_{6}{)}_{1}& ({b}_{8}{)}_{1}& ({b}_{3}{)}_{1}& ({b}_{1}{)}_{1}& ({b}_{9}{)}_{1}\end{array}\right]\left[\begin{array}{l}\varpi ({z}_{2}{)}_{0}\\ \varpi (\theta {)}_{0}\\ \varpi (f{)}_{1}\\ \varpi ({z}_{2}{)}_{1}\\ \varpi ({z}_{3}{)}_{1}\end{array}\right]\\&\quad+\,\left[\begin{array}{lllll}-h/2& 0& 1& 0& 0\\ -1& 0& 0& -h/2& 0\\ 0& -1& 0& 0& -h/2\\ ({a}_{6}{)}_{J-2}& ({a}_{8}{)}_{J-2}& ({a}_{3}{)}_{J-2}& ({a}_{1}{)}_{J-2}& ({a}_{9}{)}_{J-2}\\ ({b}_{6}{)}_{J-2}& ({b}_{8}{)}_{J-2}& ({b}_{3}{)}_{J-2}& ({b}_{1}{)}_{J-2}& ({b}_{9}{)}_{J-2}\end{array}\right]\left[\begin{array}{l}\varpi ({z}_{2}{)}_{J-2}\\ \varpi (\theta {)}_{J-2}\\ \varpi (f{)}_{J-1}\\ \varpi ({z}_{2}{)}_{J-1}\\ \varpi ({z}_{3}{)}_{J-1}\end{array}\right]=\left[\begin{array}{l}({r}_{1}{)}_\frac{1}{2}\\ ({r}_{2}{)}_\frac{1}{2}\\ ({r}_{3}{)}_\frac{1}{2}\\ ({r}_{4}{)}_\frac{1}{2}\\ ({r}_{5}{)}_\frac{1}{2}\end{array}\right].\end{aligned}$$

That is

67 $$[{B}_{J}][{\varpi }_{J-1}]+[{A}_{J}][{\varpi }_{J}]=[{r}_{J}].$$

### Stage 5: block-elimination process

Finally, in linearized finite-difference equations is obtaining the coefficient matrix known as the tridiagonal block matrix. Formulas (40)–(65) can be written as,

68 $$R\varpi =p,$$

where

69 $$R=\left[\begin{array}{llllll}{A}_{1}& {C}_{1}& & & & \\ {B}_{2}& {A}_{2}& {C}_{2}& & & \\ & \ddots & \ddots & \ddots & & \\ & & \ddots & \ddots & \ddots & \\ & & & {B}_{J-1}& {A}_{J-1}& {C}_{J-1}\\ & & & & {B}_{J}& {A}_{J}\end{array}\right],\varpi =\left[\begin{array}{l}{\varpi }_{1}\\ {\varpi }_{2}\\ \vdots \\ {\varpi }_{j-1}\\ {\varpi }_{j}\end{array}\right],p=\left[\begin{array}{l}({r}_{1}{)}_{j-\frac{1}{2}}\\ ({r}_{2}{)}_{j-\frac{1}{2}}\\ \vdots \\ ({r}_{J-1}{)}_{j-\frac{1}{2}}\\ ({r}_{J}{)}_{j-\frac{1}{2}}\end{array}\right].$$

Here $R$ signifies the $J\times J$ block-tridiagonal array with each block size of $5\times 5$, whereas, $\varpi$ and $p$ are column vectors of order $J\times 1$. The LU factorization technique is now useful to discover the solution of $\varpi$.

## Skin friction $({{\varvec{C}}}_{{\varvec{f}}})$ and Nusselt number $({\varvec{N}}{{\varvec{u}}}_{{\varvec{x}}})$

The expression regarding $({C}_{f})$ and $(N{u}_{x})$ are bestowed by (See for example Khan et al.^56^)

70 $${C}_{f}=\frac{{\tau }_{w}}{{\rho }_{f}{U}_{w}^{2}}, N{u}_{x}=\frac{x{q}_{w}}{{k}_{f}({T}_{w}-{T}_{\infty })}$$

here ${\tau }_{w}$ and ${q}_{w}$ represents stress as well as heat flux at the wall bestowed by

71 $${\tau }_{w}={\left(\left({\mu }_{nf}+\frac{1}{\stackrel{\sim }{\beta }{\varsigma }^{*}}\right)\frac{\partial u}{\partial y}-\frac{1}{6\stackrel{\sim }{\beta }{\varsigma }^{{*}^{3}}}{\left(\frac{\partial u}{\partial y}\right)}^{3}\right)}_{y=0}, {q}_{w}=-{k}_{nf}\left(1+\frac{16}{3}\frac{{\sigma }^{*}{T}_{\infty }^{3}}{{\kappa }^{*}{\nu }_{f}(\rho {C}_{p}{)}_{f}}\right){\left(\frac{\partial T}{\partial y}\right)}_{y=0}.$$

Using similarity transformations (12), above

72 $${C}_{f}R{e}_{x}^\frac{1}{2}=\left[\left(\frac{1}{(1-\phi {)}^{2.5}}+\omega \right)f^{\prime\prime}(0)-\frac{\omega\Delta }{3}(f^{\prime\prime}(0){)}^{3}\right], N{u}_{x}R{e}_{x}^{-\frac{1}{2}}=-\frac{{k}_{nf}}{{k}_{f}}\left(1+{N}_{r}\right)\theta ^{\prime}(0).$$

where, $R{e}_{x}=\frac{{U}_{w}x}{{\nu }_{f}}$ is the local Reynolds number.

## Entropy generation analysis

Generally speaking, MHD and porous media amplify entropy. Jamshed and Aziz^39^ defined entropy generation expression mentioned below.

73 $${E}_{G}=\frac{{k}_{nf}}{{T}_{\infty }^{2}}\left\{{\left(\frac{\partial T}{\partial y}\right)}^{2}+\frac{16}{3}\frac{{\sigma }^{*}{T}_{\infty }^{3}}{{\kappa }^{*}{\nu }_{f}(\rho {C}_{p}{)}_{f}}{\left(\frac{\partial T}{\partial y}\right)}^{2}\right\}+\frac{{\mu }_{nf}}{{T}_{\infty }}{\left(\frac{\partial u}{\partial y}\right)}^{2}+\frac{{\sigma }_{nf}{B}^{2}(t){u}^{2}}{{T}_{\infty }}+\frac{{\mu }_{nf}{u}^{2}}{k{T}_{\infty }}.$$

The first term depicts the transfer of heat irreversibility. The second term in the entropy expression indicates fluid friction and MHD as well as porous media effects are given at the end, respectively. The dimension-less expression regarding entropy generation $NG$ is manifested by (for instance:^57–59^

74 $$NG=\frac{{{T}_{\infty }}^{2}{c}^{2}{E}_{G}}{{k}_{f}{\left({T}_{w}-{T}_{\infty }\right)}^{2}}.$$

Using similarity transformations (12), above

75 $$NG=Re\left[{\phi }_{5}(1+Nr){\theta ^{\prime}}^{2}+\frac{1}{{\phi }_{1}}\frac{Br}{\Omega }\left({f^{\prime\prime}}^{2}+{\phi }_{1}{\phi }_{4}M{f^{\prime}}^{2}+K{f^{\prime}}^{2}\right)\right],$$

Here $Re=\frac{{U}_{w}{c}^{2}}{{\nu }_{f}x}$ is the Reynolds number, $Br=\frac{{\mu }_{f}{U}_{w}^{2}}{{k}_{f}\left({T}_{w}-{T}_{\infty }\right)}$ is the Brinkman number and $\Omega =\frac{{T}_{w}-{T}_{\infty }}{{T}_{\infty }}$ is the dimensionless temperature variation.

### Code validity

The authenticity of the proposed technique was scrutinized by taking comparison with already available literature^60–63^. Table 3 shows a strong agreement with our proposed numerical scheme. It is found that the present numerical solution is accurate up to 5 significant figures. Hence, outcomes are reliable and numerically authentic.

**Table 3:** Comparison in terms of -${\theta }^{\mathrm{^{\prime}}}(0)$ for variation in $Pr$, and taking $Q=0$, $M=0$, $\phi =0$, $\Lambda =0$, $Nr=0$, $Ec=0$, $S=0$ and $Bi=0$.

$Pr$	Ref.^60^	Ref.^61^	Ref.^62^	Ref.^63^	Present
72 × 10^–2^	080,863,135 × 10^–8^	080,876,122 × 10^–8^	080,876,181 × 10^–8^	080,876,181 × 10^–8^	080,876,181 × 10^–8^
1 × 10^0^	1 × 10^0^	1 × 10^0^	1 × 10^0^	1 × 10^0^	1 × 10^0^
3 × 10^0^	192,368,259 × 10^–8^	192,357,431 × 10^–8^	192,357,420 × 10^–8^	192,357,420 × 10^–8^	192,357,420 × 10^–8^
7 × 10^0^	307,225,021 × 10^–8^	307,314,679 × 10^–8^	307,314,651 × 10^–8^	307,314,651 × 10^–8^	307,314,651 × 10^–8^
10 × 10^0^	372,067,390 × 10^–8^	372,055,436 × 10^–8^	372,055,429 × 10^–8^	372,055,429 × 10^–8^	372,055,429 × 10^–8^

## Numerical consequences and discussion

This section is devoted to studying the influence of sundry parameters like $\omega$, $K$, $\Delta$, $M$, $\phi$, $\Lambda$, $Nr$, $Bi$, $Ec$, $Q$, $S$, $Re$, $Br$ and $m$ on velocity, temperature, and entropy generation in terms of Figs. 4, 5, 6, 7, 8, 9, 10, 11, 12, 13, 14, 15, 16, 17, 18, 19, 20, 21, 22, 23, 24, 25, 26 and 27 in the case of Cu-MeOH and GO-MeOH nanofluids. Table 5 displays the distinguished physical quantities against surface drag factor as well as temperature field gradient. The parameters Standard values adjusted at $\omega =0.1$, $K=0.1$, $\Delta =0.2$, $M=0.1$, $\phi =0.2$, $\Lambda =0.3$, $Nr=0.3$, $Bi=0.2$, $Ec=0.2$, $Q=0.1$, $S=0.1$, $Re=5$, $Br=5$ and $m=3$. The material physical properties^64, 65^ are displayed in Table 4.

**Figure 4:**
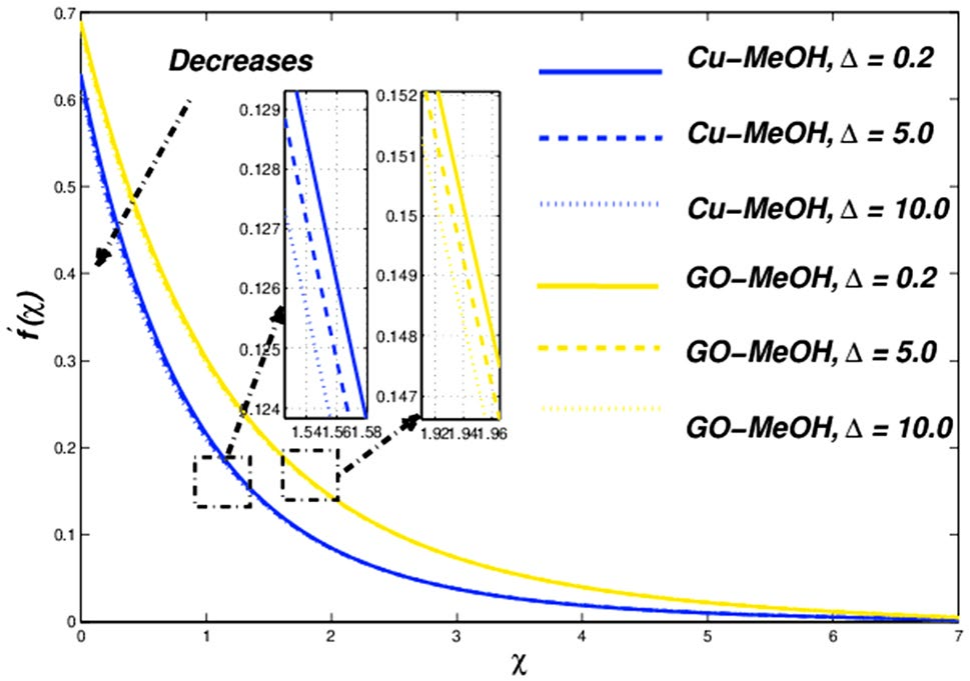
Velocity variation versus $\Delta$.

**Figure 5:**
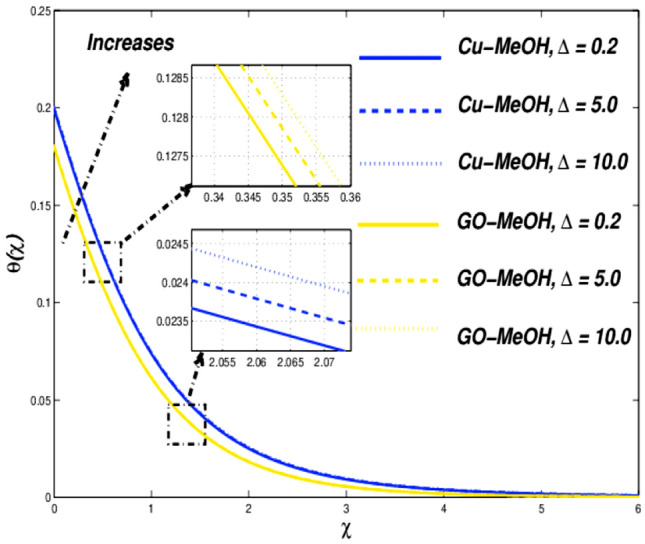
Temperature variation versus $\Delta$.

**Figure 6:**
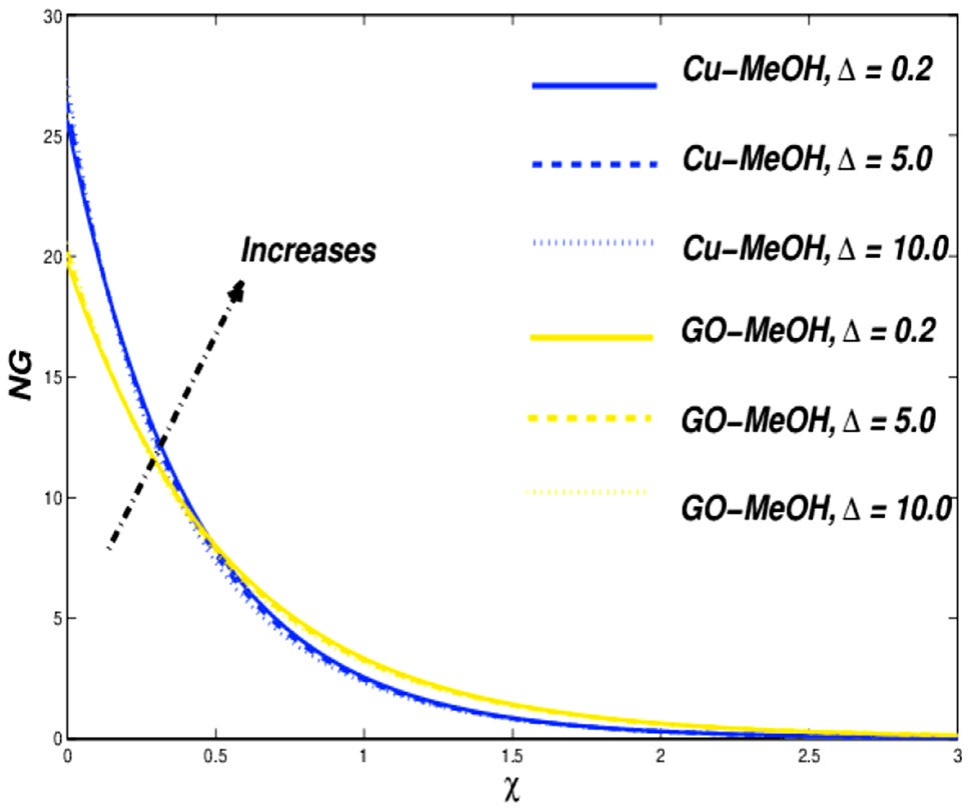
Entropy variation versus $\Delta$.

**Figure 7:**
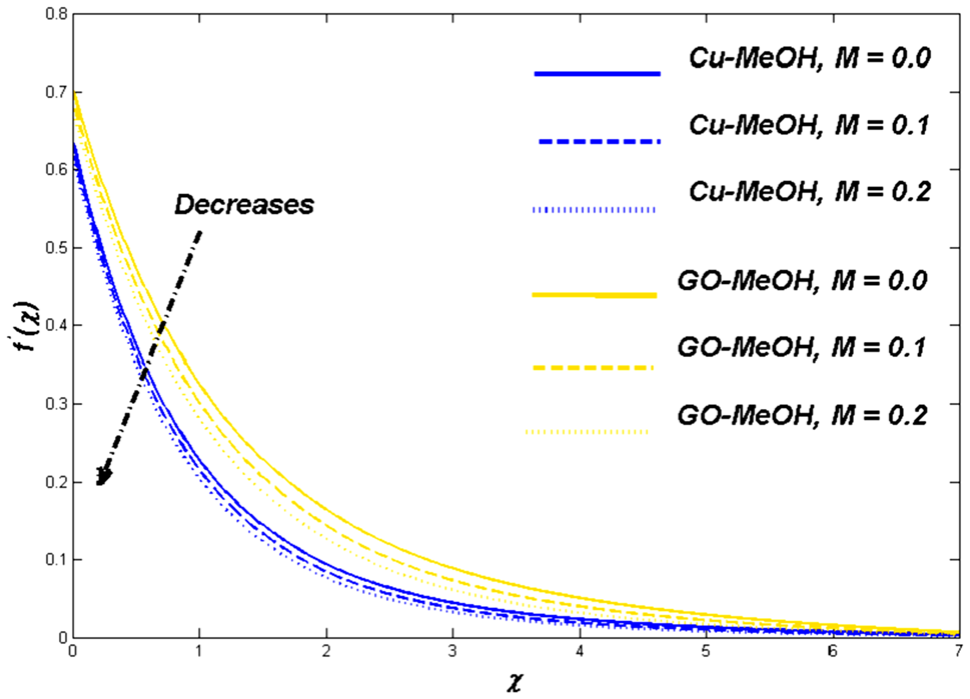
Velocity variation versus $M$.

**Figure 8:**
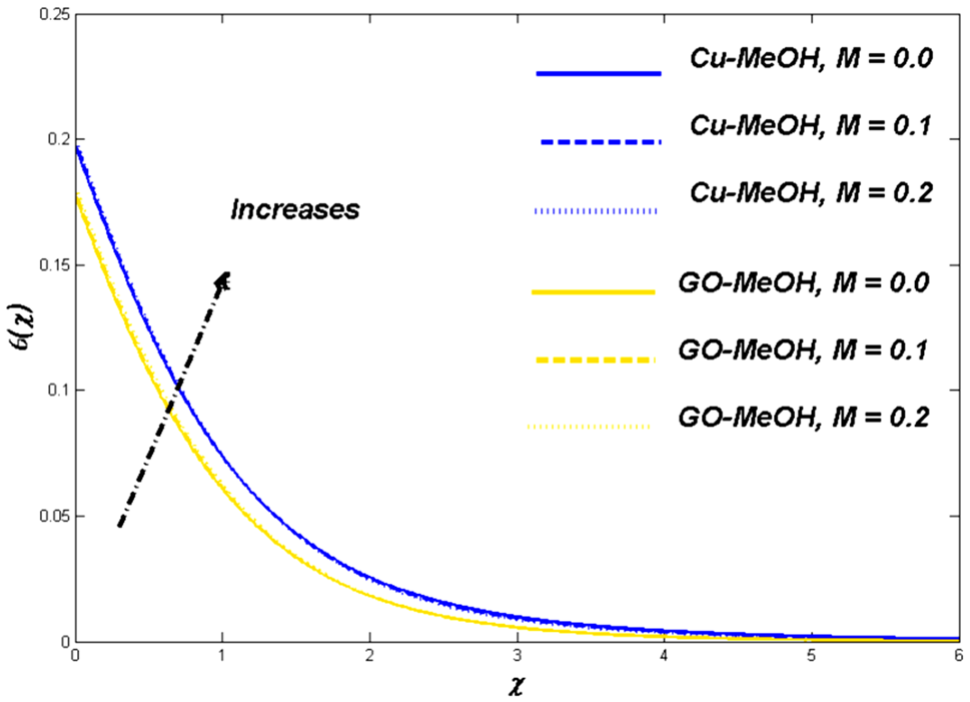
Temperature variation versus $M$.

**Figure 9:**
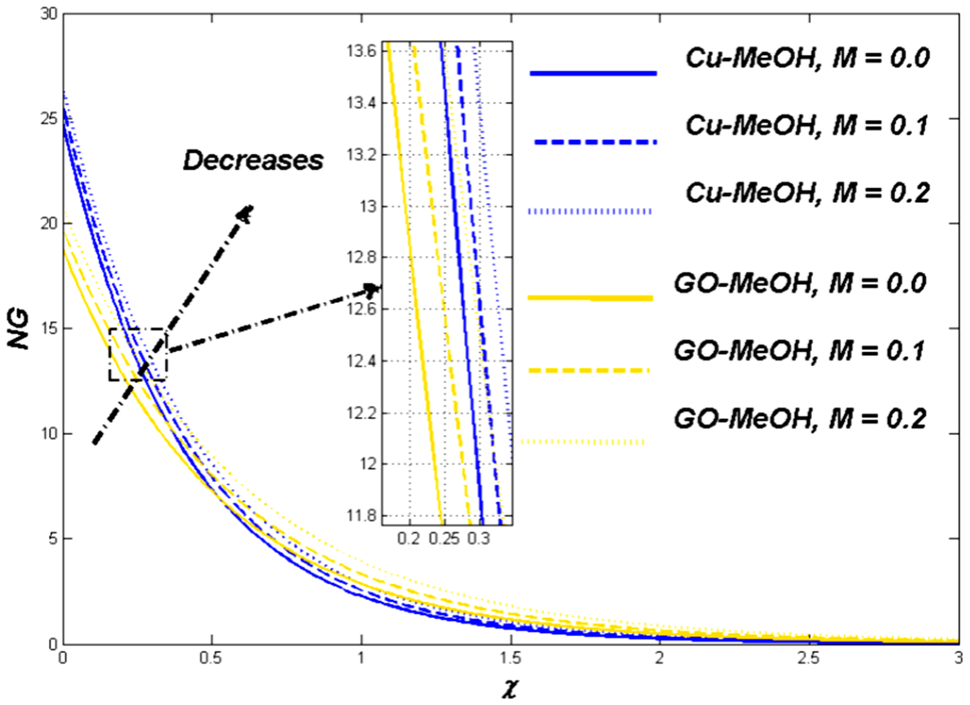
Entropy variation versus $M$.

**Figure 10:**
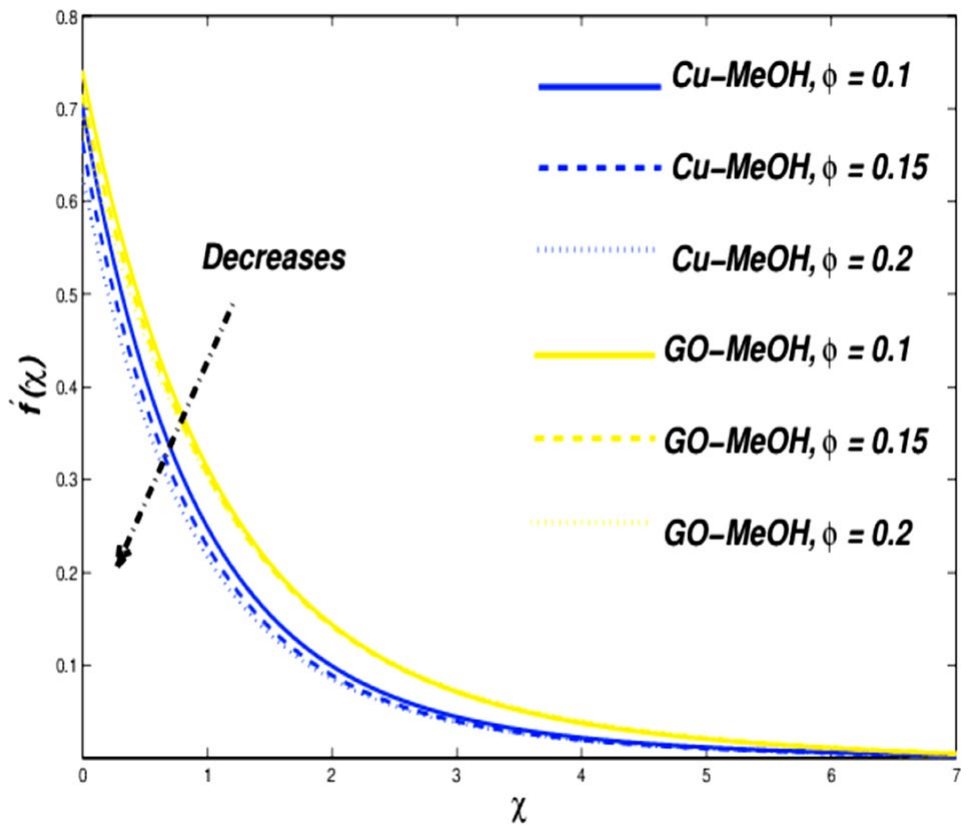
Velocity variation versus $\phi$.

**Figure 11:**
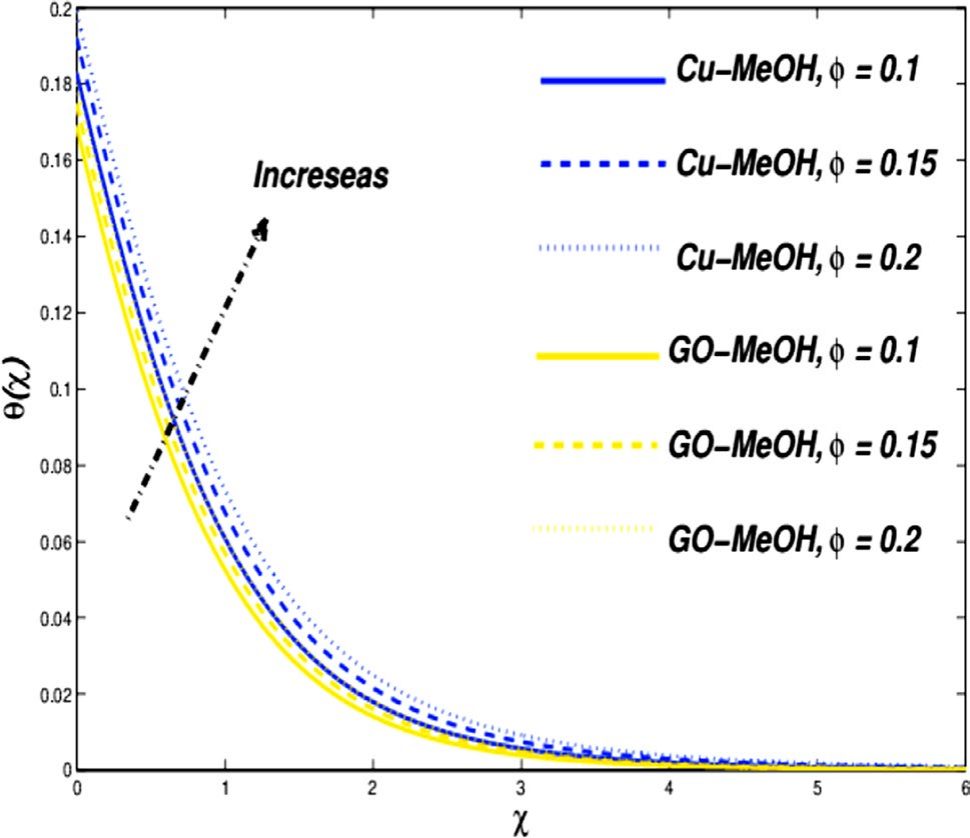
Temperature variation versus $\phi$.

**Figure 12:**
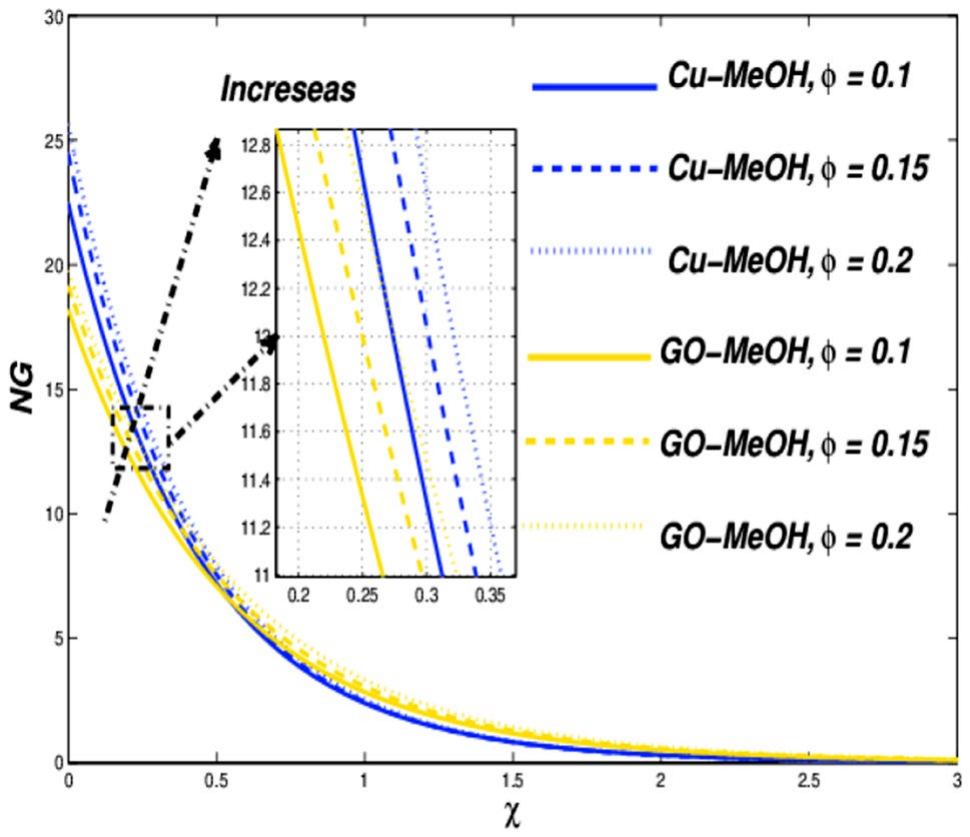
Entropy variation versus $\phi$.

**Figure 13:**
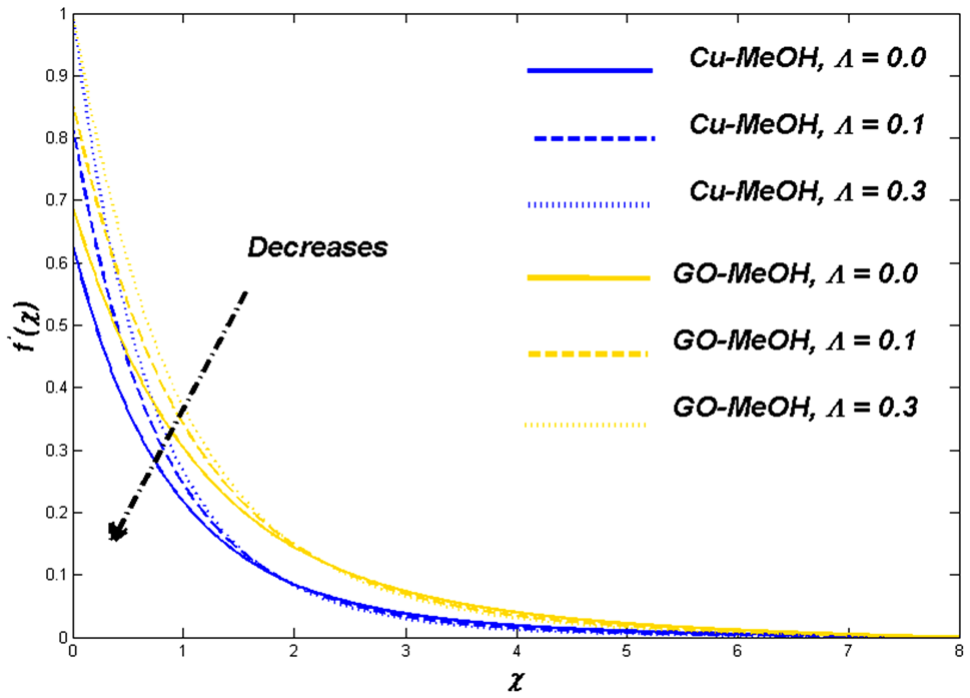
Velocity variation versus $\Lambda$.

**Figure 14:**
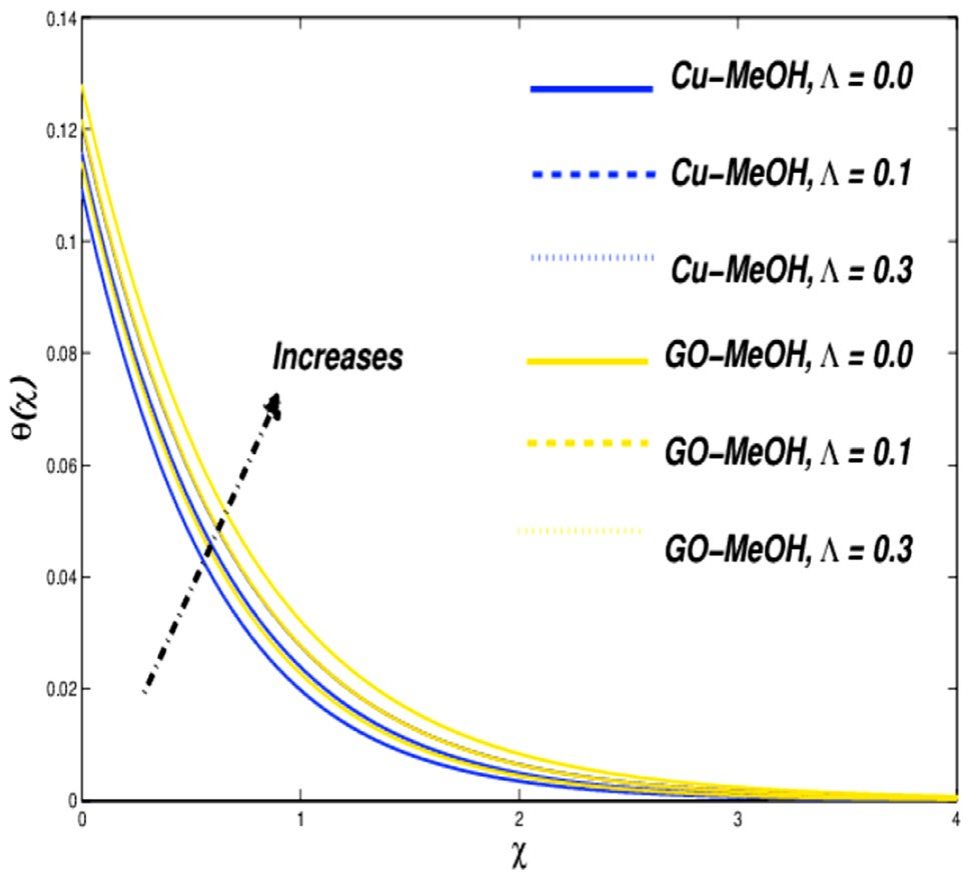
Temperature variation versus $\Lambda$.

**Figure 15:**
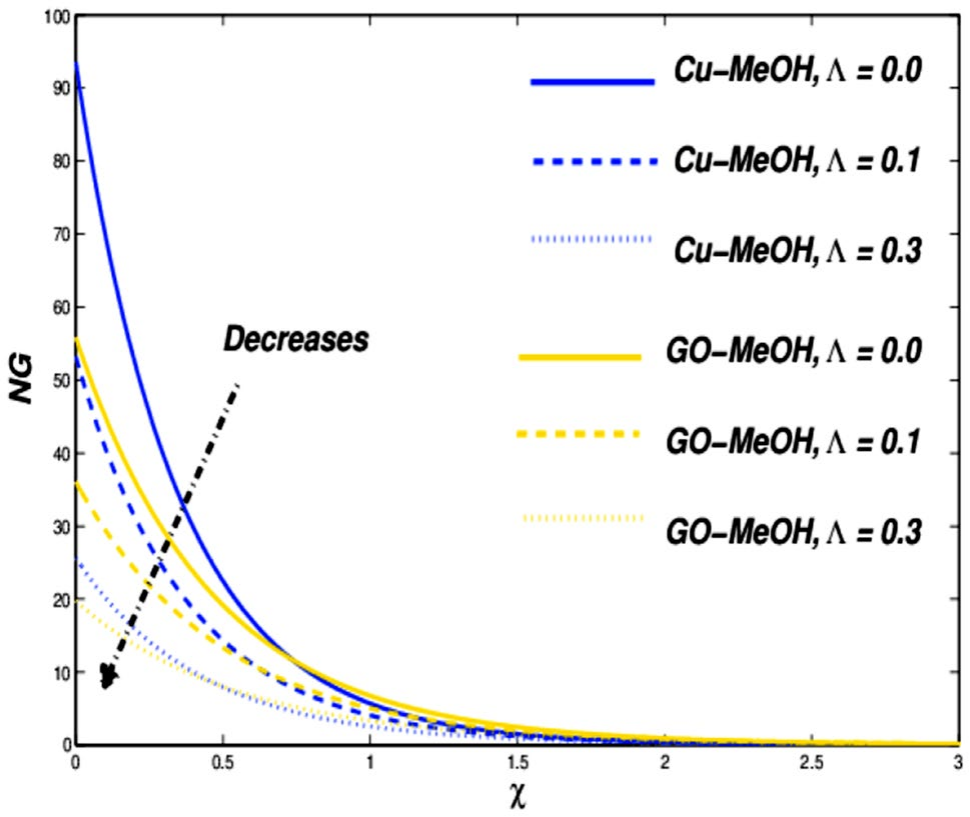
Entropy variation versus $\Lambda$.

**Figure 16:**
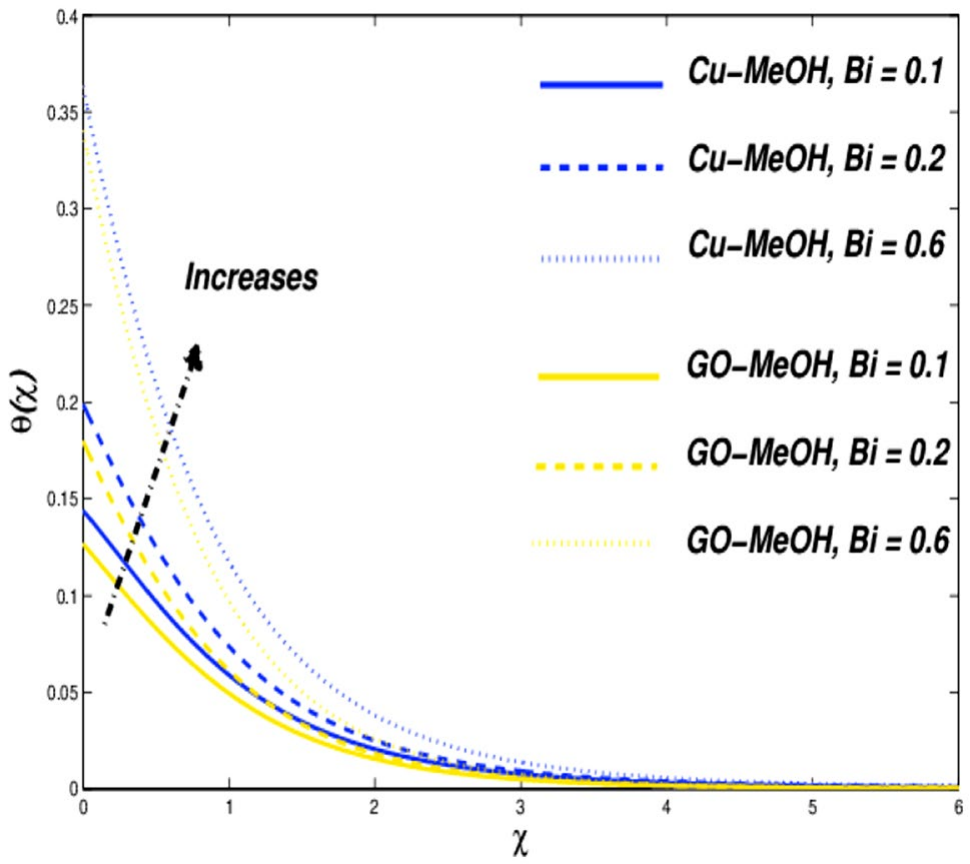
Temperature variation versus $Bi$.

**Figure 17:**
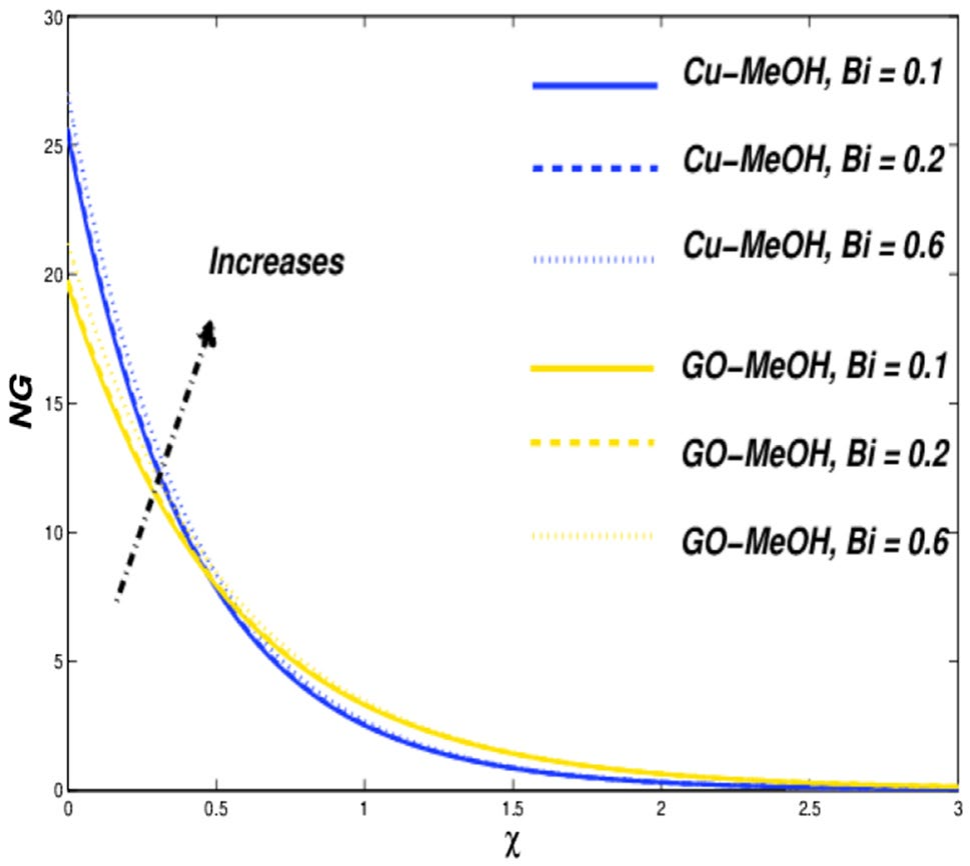
Entropy variation versus $Bi$.

**Figure 18:**
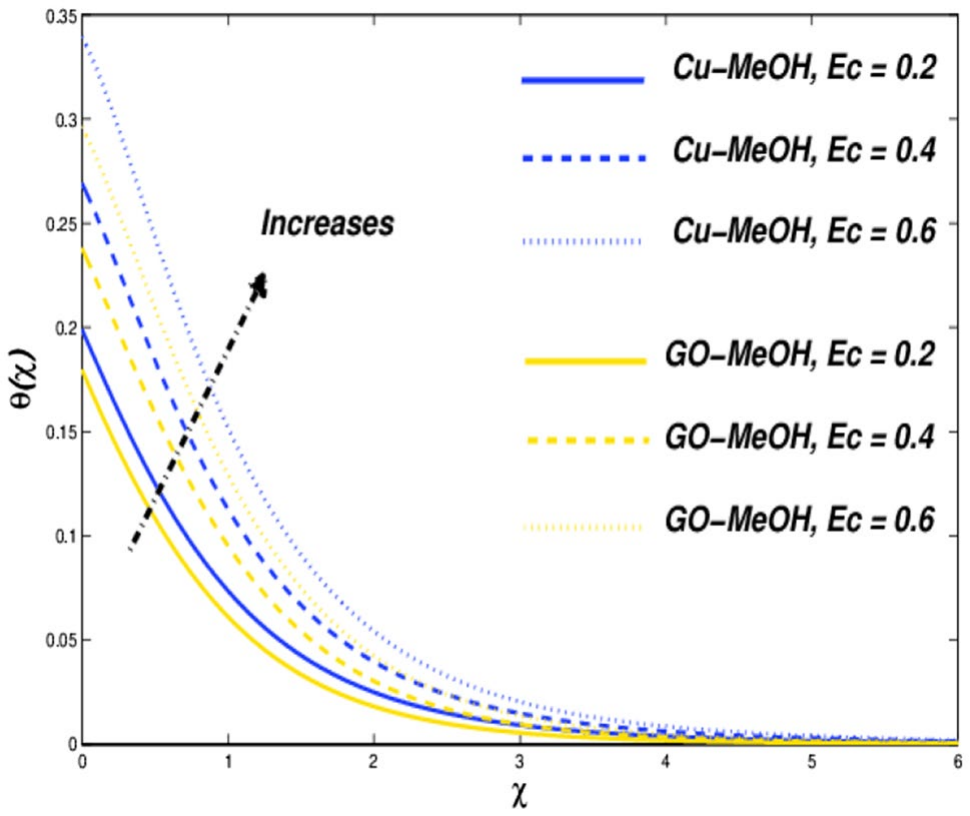
Temperature variation versus $Ec$.

**Figure 19:**
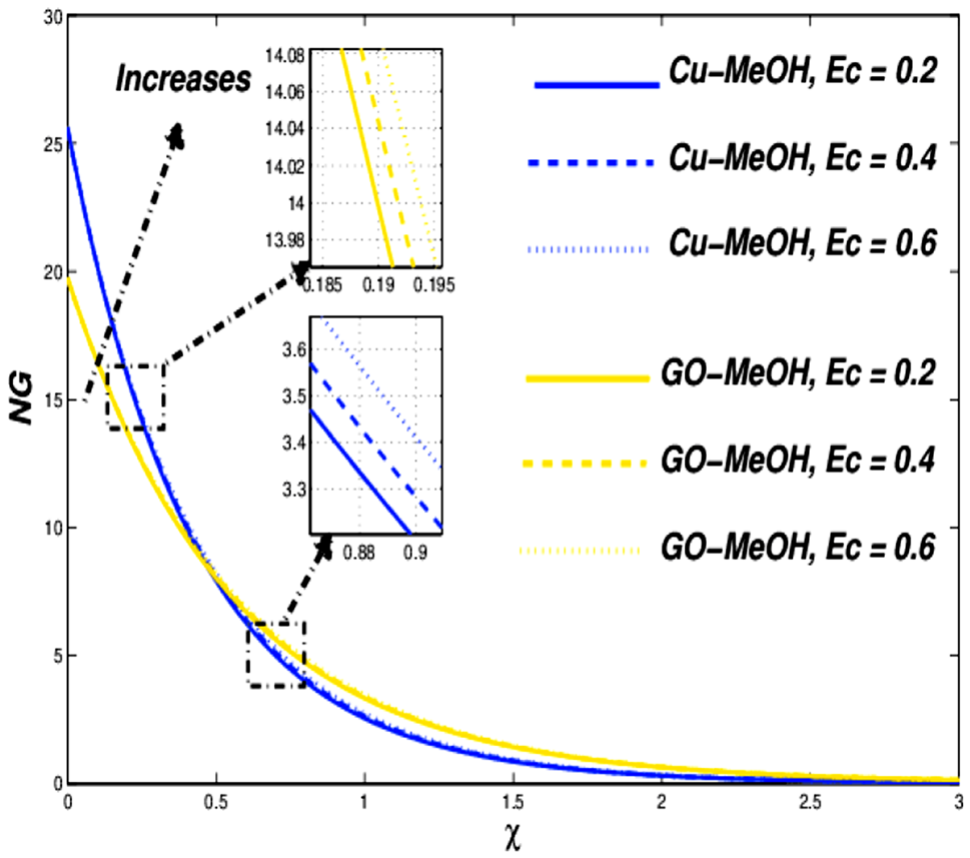
Entropy variation versus $Ec$.

**Figure 20:**
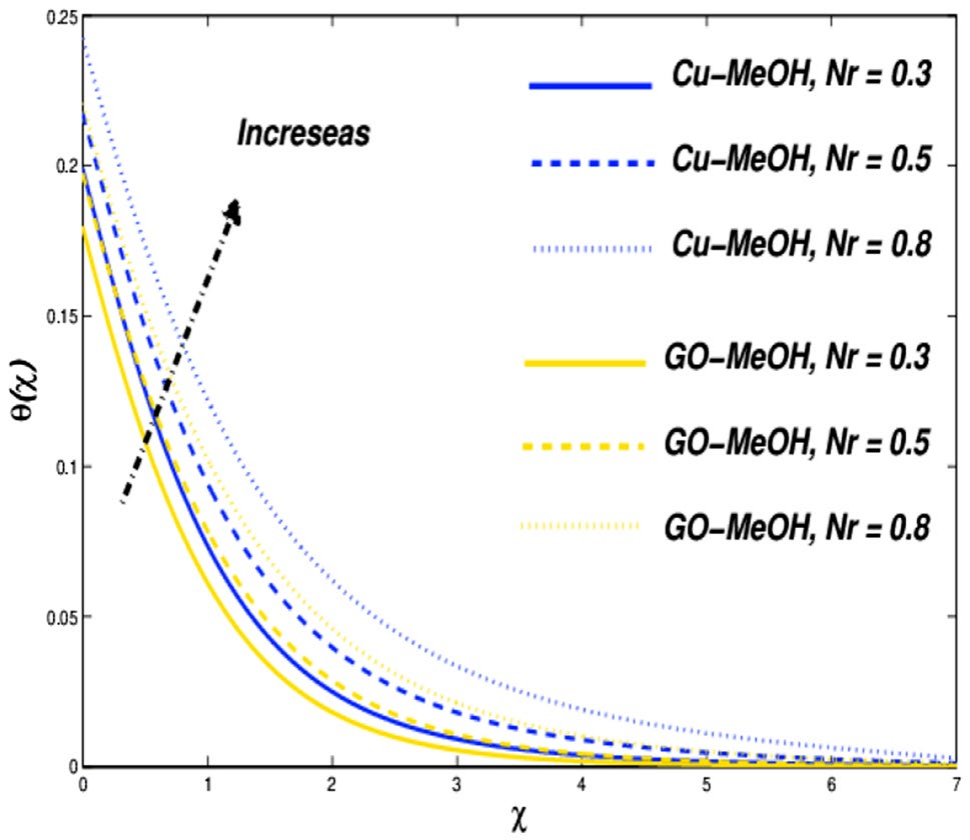
Temperature variation versus $Nr$.

**Figure 21:**
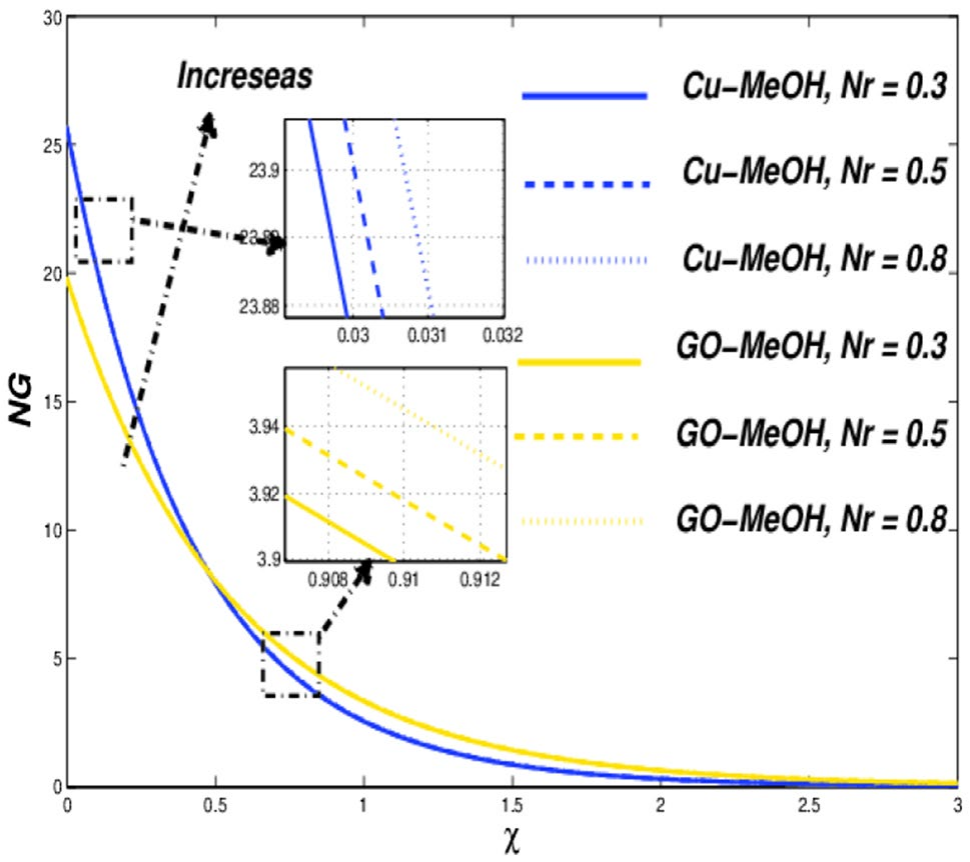
Entropy variation versus $Nr$.

**Figure 22:**
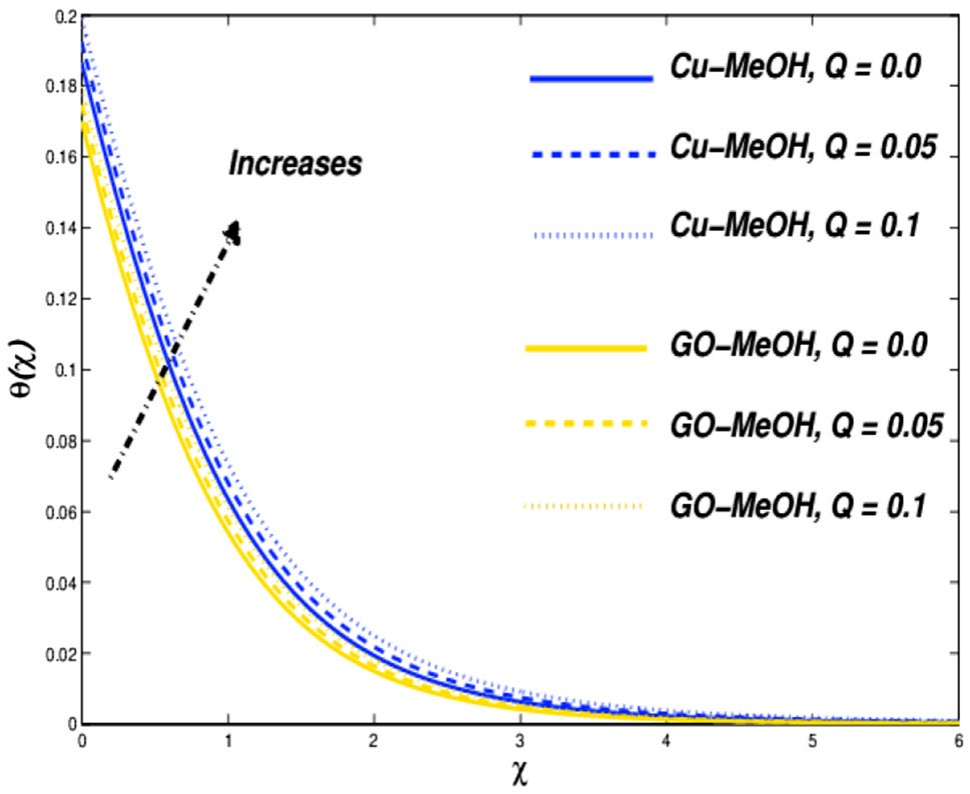
Temperature variation versus $Q$.

**Figure 23:**
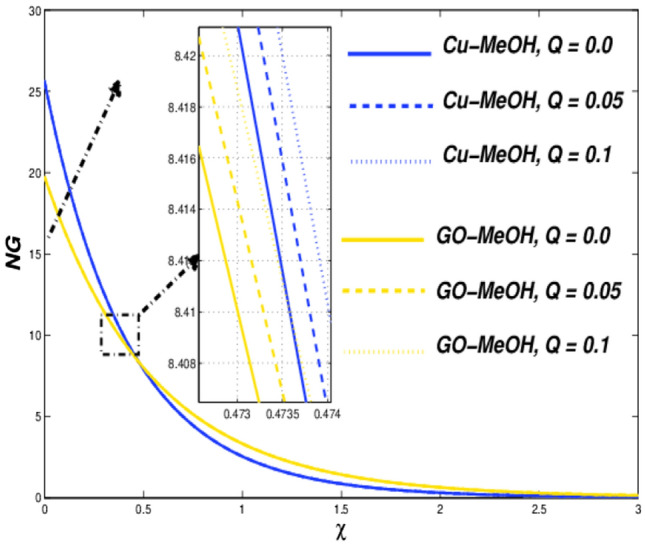
Entropy variation versus $Q$.

**Figure 24:**
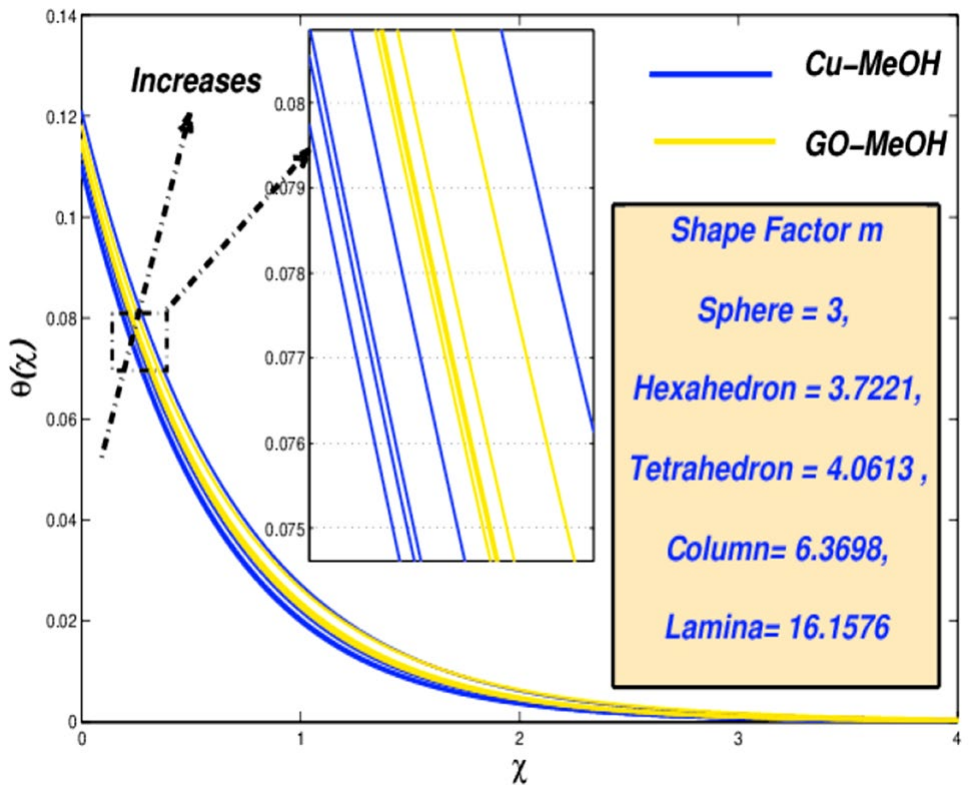
Temperature variation versus $m$.

**Figure 25:**
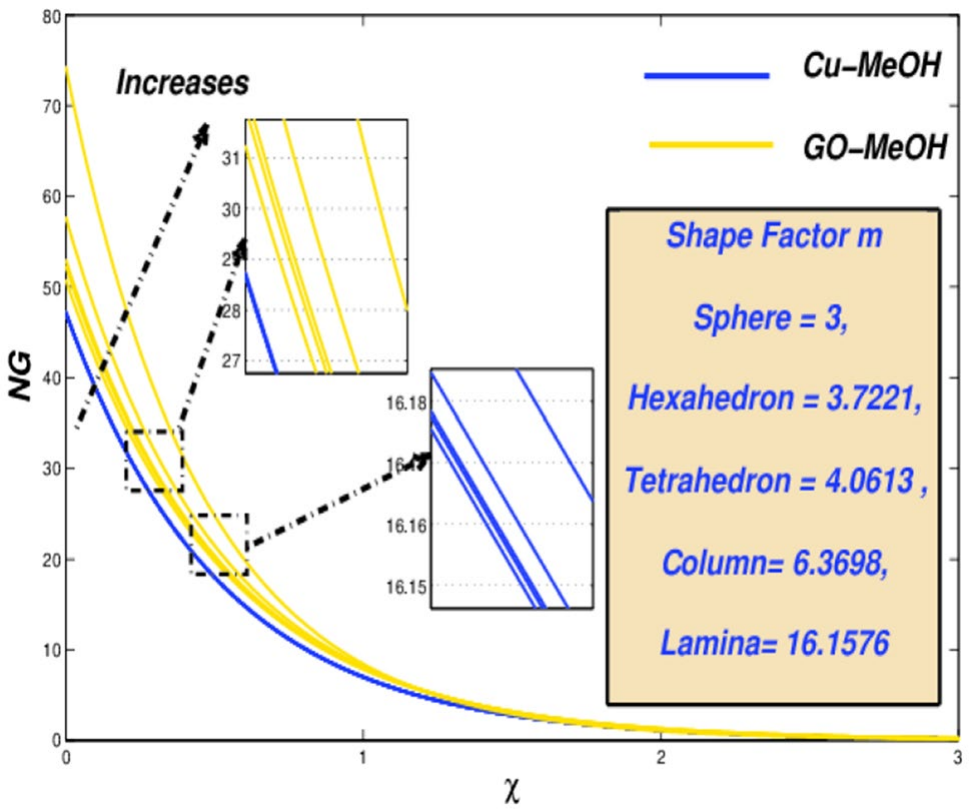
Entropy variation versus $m$.

**Figure 26:**
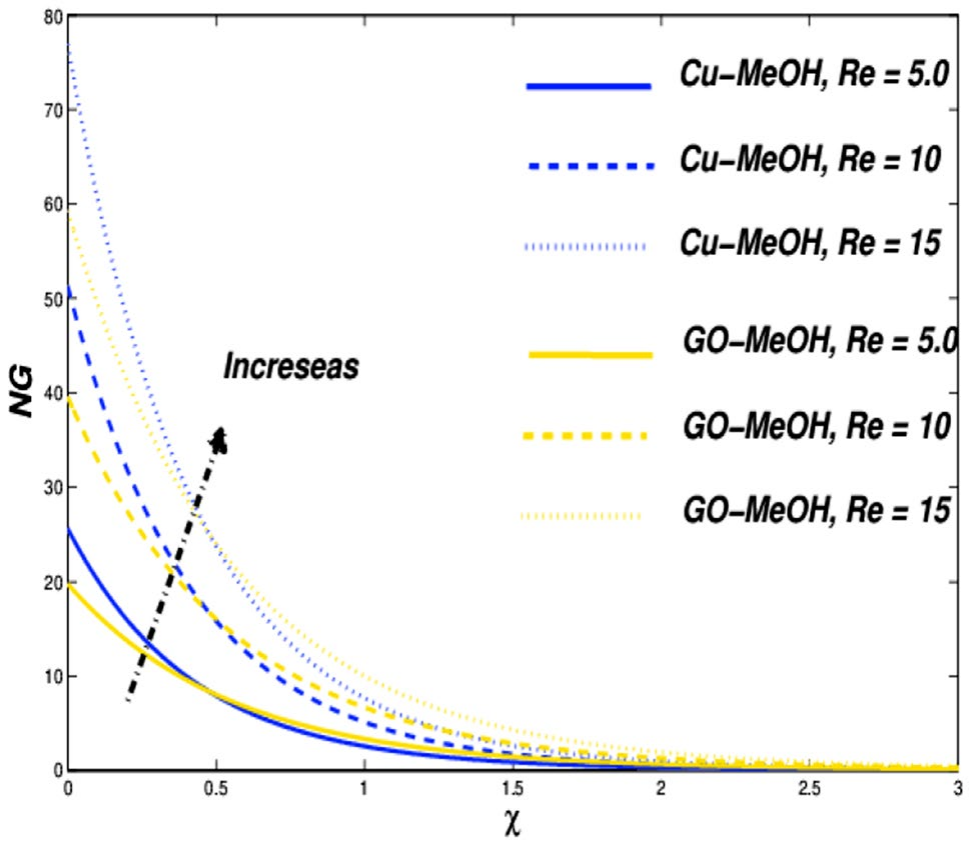
Entropy variation versus $Re$.

**Figure 27:**
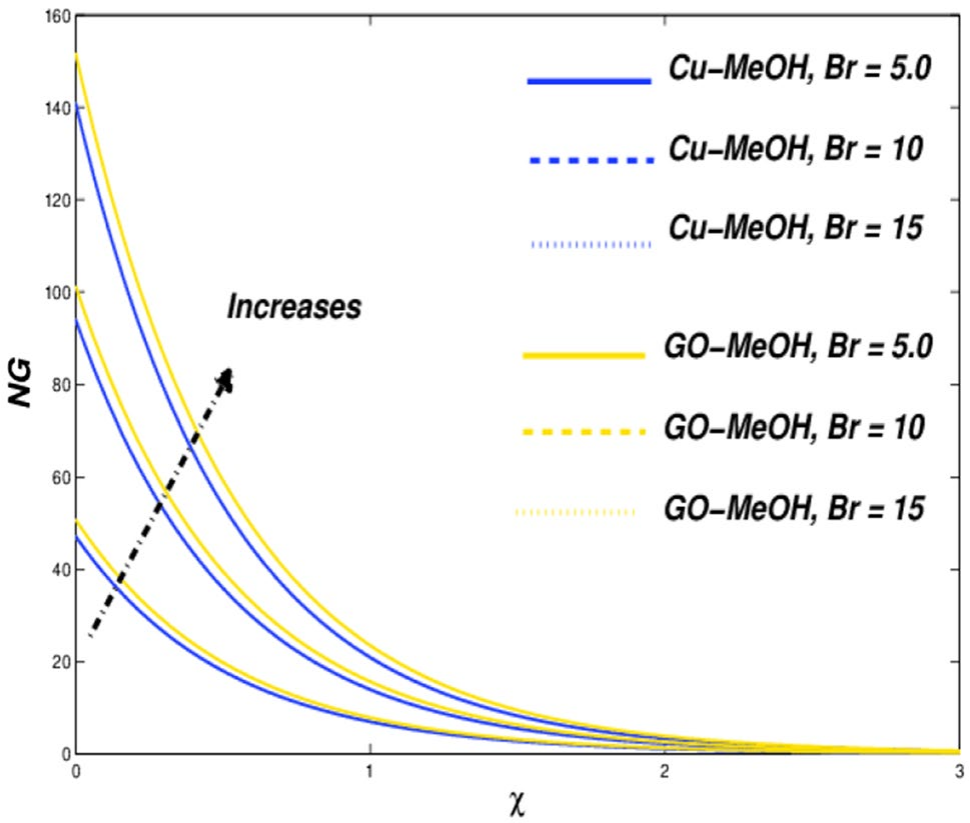
Entropy variation versus $Br$.

**Table 4:** Material properties of base fluid and nanoparticles at 293 K.

Thermophysical	$\rho (kg.{m}^{-3})$	${C}_{p} (J.k{g}^{-1})$	$k (W{m}^{-1}{K}^{-1})$
Cu	8933	385	401
MeOH	792	2545	0.2035
GO	1800	717	5000

### Effect of material parameter (${\varvec{\Delta}}$)

The influence of $\Delta$ on velocity, temperature, and entropy outlines are sketched in Figs. 4, 5 and 6 for the case of diverse values of $\Delta =\mathrm{0.2,5.0,10.0}$ at $\phi =0.2$. It is quite evident that enlargement in $\Delta$ lessens the viscosity of the fluid and diminishes fluid motion as sketched in Fig. 4. This trend assures our numerical scheme’s authenticity. Moreover, a positive variation in $\Delta$ depreciates the fluid velocity and shows a decrement in fluid motion. Incremental change in fluid viscosity depreciates yield stress. In the case of distinguished nanofluids, (when $\Delta =0.2$) the momentum boundary layer of GO-MeOH nanofluid is heavier than the Cu-MeOH nanofluid. The upsurge in nanofluid temperature is seen in Fig. 5 for magnification in $\Delta$. Heat transport rate falls in the fluid stream since an incremental change in elasticity stress. Figure 6 shows that magnification in $\Delta$ improves overall system entropy. It is expected that a magnification $\Delta$ upsurges fluid viscosity which ultimately retards the fluid motion and enlarges the temperature of the fluid and entropy phenomenon. Entropy amplifies due to a decrement in the heat transfer rate. Increment change in $\Delta$ depreciates available energy amount.

### Magnetic parameter (${\varvec{M}}$) impact

Figures 7, 8 and 9 demonstrate the influence of magnetic field $M=\mathrm{0.0,0.1,0.2}$ on temperature distribution as well as entropy generation field. Lorentz forces which are resistive forces generate a result of the electrical field in the occurrence of the magnetic field. Lorentz forces diminish the fluid velocity and thickness in terms of the momentum layer at the boundary. From Fig. 8 it is noted that the $M$ is inversely related to fluid density, so a positive variation in $M$ amplifies the boundary layer’s temperature. Table 5 validate that the Nusselt number lessens but the drag coefficient amplifies as a result of positive change in $M$. Figure 9 displayed the fact the overall entropy booms owing to an incremental change in a magnetic field. A magnification in magnetic field strength urging the fluid's speed to slow down and produces more heat, which furthermore elevates the entropy phenomenon.

**Table 5:** Values of skin friction $={C}_{f}R{e}_{x}^\frac{1}{2}$ and Nusselt number $={Nu}_{x}R{e}_{x}^{\frac{-1}{2}}$ for $Pr=7.38$.

$\omega$	$K$	$\Delta$	$M$	$\phi$	$\Lambda$	$Nr$	$Bi$	$Ec$	$Q$	$S$	${C}_{f}R{e}_{x}^\frac{1}{2}$ Cu-MeOH	${C}_{f}R{e}_{x}^\frac{1}{2}$ GO-MeOH	$N{u}_{x}R{e}_{x}^{\frac{-1}{2}}$ Cu-MeOH	$N{u}_{x}R{e}_{x}^{\frac{-1}{2}}$ GO-MeOH
0.1	0.1	0.2	$0.1$	0.2	0.3	0.3	0.2	0.2	0.1	0.1	1.0876	1.3266	0.1188	0.1201
0.3											1.1635	1.3835	0.1202	0.1286
0.5											1.3987	1.4697	0.1256	0.1323
	0.0										1.0720	1.3051	0.1228	0.1315
	0.1										1.0876	1.3266	0.1188	0.1201
	0.2										1.0995	1.3320	0.1019	0.1118
		0.2									1.0876	1.3266	0.1188	0.1201
		5.0									1.0801	1.2354	0.1124	0.1182
		10.0									1.0506	1.2195	0.1083	0.1110
			0.0								1.0520	1.2467	0.1323	0.1478
			0.1								1.0876	1.3266	0.1188	0.1201
			0.2								1.1043	1.3361	0.1097	0.1135
				0.1							0.9301	0.0791	0.1388	0.1401
				0.15							1.0532	1.2354	0.1230	0.1356
				0.2							1.0876	1.3266	0.1188	0.1201
					0.0						2.0188	2.4365	0.1272	0.1301
					0.1						1.6103	1.8115	0.1223	1.1250
					0.3						1.0876	1.3266	0.1188	0.1201
						0.3					1.0876	1.3266	0.1188	0.1201
						0.5					1.0876	1.3266	0.1243	0.1323
						0.8					1.0876	1.3266	0.1560	0.1601
							0.1				1.0876	1.3266	0.1119	0.1155
							0.2				1.0876	1.3266	0.1188	0.1201
							0.6				1.0876	1.3266	0.1232	0.1296
								0.2			1.0876	1.3266	0.1188	0.1201
								0.4			1.0876	1.3266	0.1101	0.1120
								0.6			1.0876	1.3266	0.1040	0.1080
									0.0		1.0876	1.3266	0.1232	0.1328
									0.05		1.0876	1.3266	0.1202	0.1271
									0.1		1.0876	1.3266	0.1188	0.1201
										0.1	1.0876	1.3266	0.1188	0.1201
										0.3	1.3201	1.4332	0.1234	0.1299
										0.4	1.4001	1.5112	0.1307	0.1350

### Nanoparticle concentration size (${\varvec{\phi}}$) Impact

Figures 10 and 11 reflect the impact of $\phi =\mathrm{0.1,0.15,0.2}$ on fluid velocity along with temperature field as well. It is noteworthy that a positive variation in $\phi$ makes the fluid dense to flow over the surface which lessens the fluid velocity and thickness of the momentum boundary layer as well. It is observed that the addition of nanoparticles in base fluid amplifies heat transfer rate and thermal conduction phenomenon. As a result temperature of the fluid and thickness of the thermal-based boundary layer have been improved tremendously as depicted in Fig. 11. The velocity as well as and temperature in terms of $\phi$ is portrayed in Table 5. Figure 12 showed that a positive change in entropy as a result of magnification in $\phi$. This trend is similar to the effect of magnetic parameter $M$. It is obvious that the higher nanoparticle’s concentration, the greater entropy of the system.

### Slip velocity parameter (${\varvec{\Lambda}}$) impact

Figures 13 and 14 reflects the variation in velocity and temperature outlines in the case of diverse values of $\Lambda =\mathrm{0.0,0.1,0.3}$. In the case of the slip phenomenon the velocity of the fluid and sheet on which the fluid flow is not identical. Moreover, a stretching phenomenon between surface and fluid diminishes which retard the fluid flow motion and improves the heat transfer rate and temperature distribution inside the fluid well because velocity, as well as temperature distribution, are inversely linked with each other. The slip impact on expandable sheet velocity is displayed in Fig. 13. This phenomenon happens because it reduces the stretching effect retards fluid velocity. Figure 14 shows that $\Lambda$ is inversely related to temperature. Amplification in $\Lambda$ diminishes heat transfer but elevates temperature at the boundary. Overall entropy booms owing to an amplification in the temperature because the slip phenomenon depreciates the friction effect which ultimately magnifies the temperature as well as entropy as displayed in Fig. 15.

### Biot number (${\varvec{B}}{\varvec{i}}$), and Eckert number (${\varvec{E}}{\varvec{c}}$) impact

Figures 16, 17, 18 and 19 are planned to reflect the influence of $Bi$ and $Ec$ on temperature as well as entropy fields likewise. Convection in terms of heat transfer from the boundary towards the fluid is getting better and better owing to an amplification in the values of $Bi$. The temperature as well as thickness in terms of a thermal layer at the boundary booms as a result of enrichment in $Bi$ (Fig. 16). No significant change is reported in the case of the velocity field in the case of a positive variation in $Bi$. Figure 18 sketches the change in temperature field for the case of the diverse values of $Ec=\mathrm{0.2,0.4,0.6}$. Greater $Ec$, a ratio of kinetic energy to enthalpy difference. Molecules collide more randomly as a result of an increment in $Ec$ because the kinetic energy amplifies the molecules' friction and internal heat generation capacity which elevates the heat transfer phenomenon in a temperature field as portrayed in Fig. 18. Figures 17 and 19 it is quite evident that a substantial amplification in parameters $Bi$ as well as $Ec$ provides substantial heat to the fluid which upsurges temperature phenomenon and entropy phenomenon as well. In the case of $\chi =0.3$, the entropy showed a cross-over point. Entropy amplifies and declined before as ell as after that point.

### Thermal radiative (${\varvec{N}}{\varvec{r}}$) and heat source parameters (${\varvec{Q}}$) impact

Figure 20 shows the impact of $Nr$ on the temperature distribution field for various values of $Nr=\mathrm{0.3,0.5,0.8}$. Thermal radiation is used where a large temperature difference is required like combustion reactions, nuclear fusions, ceramic productions, etc. In the presence of $Nr$ temperature as well heat transfer phenomenon escalates by the virtue of amplification in $Nr$. It is quite interesting that more is generated inside the fluid on the behalf of augmentation in the heat source $Q$ which ultimately makes a pathway for magnification in the temperature field as sketched in Fig. 22. Figures 21 and 23 exhibit the influence of $Nr$ as well as $Q$ parameters on entropy profile. It is quite clear that in the case of $\chi =0.3$, the entropy outline depicts incompatible facts. Entropy of the system amplifies before that point while depreciates after that point. Physically, the crossover point is a sign for effective modification of the thermal system. We can say that $\chi =0.3$ has situated nearby the sheet and the entropy always upsurges close to the boundary sheet and depreciates in the case of away from the surface.

### Nanoparticle shapes factor (${\varvec{m}}$) impact

It is quite important that amplification or decrement in heat transfer rate phenomenon solely relies on the values of nanoparticles shape factor $m$. The $m$ effect on temperature as well as entropy fields is displayed in Figs. 24 and 25 by considering five different shapes of nanoparticles. $m=3.0$(sphere), 3.7221 (hexahedron), 4.0613 (tetrahedron), 6.3698 (column), 16.1576 (lamina) for distinguished shapes are represented in Table 2. From Fig. 24 it is observed that nanofluid temperature upsurges by the virtue of an improvement in $m$. Temperature of the fluid is getting lower at $m=3$ spherical-shaped type nanoparticles. The sphere occupies a large superficial zone and booms heat transmission rate from the sheet surface towards fluid inside. It is noted from Fig. 25, entropy $m$ escalates. The system's entropy is getting lower and lower for sphere structure shape-particles as the heat trick inside the scheme is getting smallest.

### Reynolds (${\varvec{R}}{\varvec{e}}$) and Brinkman numbers (${\varvec{B}}{\varvec{r}}$) influences

Lastly, the impacts of $Re=5.0, 10.0, 15.0$ and $Br=5.0, 10.0, 15.0$ on entropy generation are presented. It is noteworthy that the inertial forces topple viscous forces in the case of magnification in $Re$ which furthermore enhances the overall entropy of the thermal system shown in Fig. 26. Figure 27 sketches the $Br$ effect on entropy. In the case of augmentation in $Br$, heat dissipates more quickly as compared to the conduction phenomenon at the surface, which moreover amplifies entropy of the system. The results are quite reliable in comparison with Abbas et al.^66^, who reported similar results.

#### Impact of pertinent dimensionless parameters on skin friction coefficient $\left({{\varvec{C}}}_{{\varvec{f}}}\right)$ and Nusselt number $\left({\varvec{N}}{{\varvec{u}}}_{{\varvec{x}}}\right)$

The influence of sundry dimensionless parameters on $\left({C}_{f}\right)$ and $\left(N{u}_{x}\right)$ are presented in the table enumerated underneath.

## Conclusions

Computational surveys of boundary-layer flow for Cu and GO methanol-based nanofluids were achieved over a permeable elongating surface. This research considered MHD, porous medium, viscous dissipative, thermal radiative, Joule-heating, and particle shapes with Keller box methods help. Significance of the effects of different dimensionless parameters against velocity, Temperature, and entropy profiles are displayed in terms of figures. The ${C}_{f}$ as well as $N{u}_{x}$ for diverse amounts of sundry factors are portrayed in the form of a table. Some pertinent concluding observations from the present study are enumerated underneath.

1. Velocity profile owing to amplification in $\Delta$ and $\phi$.
2. Temperature profile increased the function of parameters $\Delta$, $K$, $M$, $\phi$, $Nr$, $Bi$, $Q$ and $Ec$ whereas reduced parameters $S>0$.
3. Amplification in nanoparticles concentration $\phi$ guides an improvement in temperature and thermal boundary layer thickness.
4. The GO-MeOH nanofluid is better in terms of thermal conduction instead of Cu-MeOH nanofluid.
5. The heat transport rate is more significant for the lesser number of shape factors.
6. Overall systems entropy depreciates by the virtue of magnification in slip parameter.
7. Lamina-shaped particles deliver more heat at the boundary layer, while the temperature is getting lower for the case of spherical-shaped nanoparticles.

## Abbreviations

$B$: Magnetic field parameter
$$Br$$: Brinkman number
$c$: Stretching rate parameter
${C}_{f}$: Surface drag coefficient
$${C}_{p}$$: Specific heat ($${\text{KJ}}\,{\text{g}}^{ - 1} \,{\text{K}}^{ - 1}$$)
$$\rho {C}_{p}$$: Heat capacity ($${\text{KJ}}^{ - 1} \,{\text{m}}^{ - 3}$$)
$${E}_{G}$$: Entropy production
$$Ec$$: Eckert number
$f(\psi )$: Nondimensional stream function
${h}_{f}$: Heat transport factor
$k$: Porosity of fluid
$K$: Porous media parameter
$${k}^{*}$$: Absorption factor
$$M$$: Magnetic field
$$Nr$$: Thermal radiative
$$Nu$$: Nusselt number
$$\mathrm{Pr}$$: Prandtl number
$$Q$$: Heat source
${q}_{r}$: Radiation heat flux
$$Re$$: Reynolds number
$S$: Mass transport parameter
$T$: Temperature of the fluid (K)
${T}_{w}$: Temperature at the wall (K)
$${T}_{\infty }$$: Ambient temperature (K)
$$t$$: Time (s)
$u,v$: Velocity components (ms^−^^1^)
$${U}_{w}$$: Surface velocity
${V}_{w}$: Permeability of sheet
$x,y$: Cartesian co-ordinates

## Greek symbols

$$\alpha$$: Thermal diffusion
$$\stackrel{\sim }{\beta },{\zeta }^{*}$$: Material constants
$$\phi$$: Nanoparticle size
$$\kappa$$: Thermal conductance
$$\sigma$$: Electrical conductance
$$\theta$$: Nondimensional temperature
$\mu$: Dynamical viscidness (kg m^−^^1^ s^−^^1^)
$$\nu$$: Kinematic viscosity (m^2^ s^−^^1^)
$$\omega ,\Delta$$: Material parameter
$\Omega$: Nondimensional temperature variant
$$\rho$$: Density (kg m^−^^3^)
${\tau }_{ij}$: Cauchy stress-tensor
${\tau }_{w}$: Drag force (kg m^−^^1^ s^−^^2^)
$$\tau$$: Dimensionless time
$$\Lambda$$: Slip velocity

## Subscripts

$$f$$: Fluid
$$nf$$: Nanofluid
$$s$$: Nanoparticles

## Superscript

$\mathrm{^{\prime}}$: Differentiation concerning to *ψ*
